# Stem cell-derived brainstem mouse astrocytes obtain a neurotoxic phenotype in vitro upon neuroinflammation

**DOI:** 10.1186/s12950-023-00349-8

**Published:** 2023-06-27

**Authors:** Caroline Lindblad, Susanne Neumann, Sólrún Kolbeinsdóttir, Vasilios Zachariadis, Eric P. Thelin, Martin Enge, Sebastian Thams, Lou Brundin, Mikael Svensson

**Affiliations:** 1 Department of Clinical Neuroscience, Karolinska Institutet, J5:20 Svensson Group, Karolinska Universitetssjukhuset Solna, SE-171 77 Stockholm, Sweden; 2 Department of Oncology-Pathology, Karolinska Institutet, Stockholm, Sweden; 3 Department of Neurology, Karolinska University Hospital, Stockholm, Sweden; 4 Department of Neurosurgery, Karolinska University Hospital, Stockholm, Sweden

**Keywords:** Embryonic stem cells, Disease modelling, Traumatic axonal injury, Ventral brainstem- or rostroventral spinal astrocytes, Astrocytes, Neuroinflammation

## Abstract

**Background:**

Astrocytes respond to injury and disease through a process known as reactive astrogliosis, of which inflammatory signaling is one subset. This inflammatory response is heterogeneous with respect to the inductive stimuli and the afflicted central nervous system region. This is of plausible importance in e.g. traumatic axonal injury (TAI), where lesions in the brainstem carries a particularly poor prognosis. In fact, astrogliotic forebrain astrocytes were recently suggested to cause neuronal death following axotomy. We therefore sought to assess if ventral brainstem- or rostroventral spinal astrocytes exert similar effects on motor neurons in vitro.

**Methods:**

We derived brainstem/rostroventral spinal astrocyte-like cells (ES-astrocytes) and motor neurons using directed differentiation of mouse embryonic stem cells (ES). We activated the ES-astrocytes using the neurotoxicity-eliciting cytokines interleukin- (IL-) 1α and tumor necrosis factor-(TNF-)α and clinically relevant inflammatory mediators. In co-cultures with reactive ES-astrocytes and motor neurons, we assessed neurotoxic ES-astrocyte activity, similarly to what has previously been shown for other central nervous system (CNS) regions.

**Results:**

We confirmed the brainstem/rostroventral ES-astrocyte identity using RNA-sequencing, immunocytochemistry, and by comparison with primary subventricular zone-astrocytes. Following cytokine stimulation, the c-Jun N-terminal kinase pathway down-stream product phosphorylated c-Jun was increased, thus demonstrating ES-astrocyte reactivity. These reactive ES-astrocytes conferred a contact-dependent neurotoxic effect upon co-culture with motor neurons. When exposed to IL-1β and IL-6, two neuroinflammatory cytokines found in the cerebrospinal fluid and serum proteome following human severe traumatic brain injury (TBI), ES-astrocytes exerted similar effects on motor neurons. Activation of ES-astrocytes by these cytokines was associated with pathways relating to endoplasmic reticulum stress and altered regulation of MYC.

**Conclusions:**

Ventral brainstem and rostroventral spinal cord astrocytes differentiated from mouse ES can exert neurotoxic effects in vitro. This highlights how neuroinflammation following CNS lesions can exert region- and cell-specific effects. Our in vitro model system, which uniquely portrays astrocytes and neurons from one niche, allows for a detailed and translationally relevant model system for future studies on how to improve neuronal survival in particularly vulnerable CNS regions following e.g. TAI.

**Supplementary Information:**

The online version contains supplementary material available at 10.1186/s12950-023-00349-8.

## Introduction

Astrocytes constitute the most abundant cell type in the central nervous system (CNS) [1], and effectuate diverse functions [2]. Following injury or disease, astrocytes undergo reactive astrogliosis (synonymous to *astrocyte reactivity*) [3–5], an evolutionary conserved response [4] that can be triggered by almost every type of CNS insult, stretching from infectious diseases, traumatic injuries, to neurodegenerative conditions [2]. Reactive astrogliosis encompass a plethora of astrocytic alterations [5], that manifest differently depending on the inductive stimuli [6]. Among these, astrocytes undergo e.g. neuroinflammatory activation [7]. These inflammatory responses are influenced by astrocytic distance from the lesion site [8], as well as the CNS region of injury or disease [9], thus contributing to further complexity and heterogeneity. This is of importance in for example traumatic brain injury (TBI), where brainstem traumatic axonal injury (TAI) is associated with poor neurological outcome [10, 11].

Early work demonstrated signs of neuroinflammation in TAI-specific experimental models [12–14]. Astroglial activation has been shown to coincide with TAI [15], even in the absence of peripheral immune cell infiltration [16]. Recently, reactive forebrain astrocytes have been demonstrated to exert neurotoxic effects, and this has been suggested to be the mechanism through which neurons succumb following axotomy [17]. Whether this also applies to the brainstem and spinal cord has been unclear, as at least some aspects of the astrocytic inflammatory response are CNS region-specific [18]. Recently, *cortical* neurons (derived from human induced pluripotent stem cells [hiPSC] and rodent primary cultures) were shown to upregulate caspase pathways when co-cultured with neurotoxic spinal cord hiPSC-astrocytes [19]. In experimental models of amyotrophic lateral sclerosis, mutated spinal astrocytes have been demonstrated to confer a motor neuron selective neurotoxic effect [20]. How brainstem and spinal cord motor neurons would respond to neurotoxic astrocyte stimulation is however yet unknown. This could be of large interest in TAI, as it could represent an unexplored secondary mechanism aggravating secondary axotomy. We therefore set out to create a translational model system of mouse stem cell-derived brainstem reactive astrocytes in co-culture with motor neurons from the same CNS niche.

## Materials and methods

All experiments were conducted in accordance with Swedish legislation, and as stipulated in the Code of Regulations of the Swedish Board of Agriculture [21]. Ethical approval was granted by the Swedish Board of Agriculture’s regional Stockholm County branch ethics committee (#9182–2018; and #N104/14). Experimental animals were housed at the Karolinska Institutet animal facility with ad libitum access to water and food on a 12 h dark-/light cycle.

### Directed differentiation of embryonic stem cells into brainstem motor neurons

Mouse embryonic stem cells (ES-cells) carrying an enhanced green fluorescent protein (eGFP) under the homeobox gene 9- (Hb9-) promotor (mouse strain origin: B6.Cg-Tg(Hlxb9-GFP)1Tmj/J) were utilized. ES-motor neurons were differentiated in accordance with Wichterle and colleagues [22]. Protocol details are provided in Supplementary Methods. In brief, ES cells were seeded in T25 flasks (VWR) coated with 0.1% gelatin (Merck Millipore) in ES medium (Supplementary Table 1). Differentiation was induced following two days of expansion (Supplementary Table 1). Two days after initiation of differentiation, resulting free-floating embryoid bodies (EBs) were supplemented with 1µM retinoic acid and 0.5 µM smoothened agonist. On day five, medium was replaced with fresh differentiation medium, supplemented with only 10 ng/mL glial cell-line derived neurotrophic factor. On day six, EBs were dissociated into single cell populations of neurons and glial progenitor cells using 0.05% trypsin (Gibco) and trituration. Cells were resuspended in either motor neuron medium (Supplementary Table 1) or as described below.

### Directed differentiation of embryonic stem cells into brainstem astrocyte-like cells

ES-derived brainstem/rostroventral spinal astrocyte-like cells (ES-astrocytes) were differentiated using previously described protocols [23, 24], that we modified. For downstream analysis, we used 96-well plates (Greiner Bio-One) coated with 100 µg/ml poly-L-ornithine hydrobromide and 1 µg/ml laminin (Supplementary Table 2). Dissociated EBs (see Directed differentiation of embryonic stem cells into brainstem motor neurons) were plated at a density of 15 * 10^6^ cells on poly-L-ornithine/laminin coated dishes (VWR) in astrocyte medium (Supplementary Table 2) supplemented with basic fibroblast growth factor (FGFb, 25 ng/mL, R&D) and epidermal growth factor (EGF, 20 ng/mL, R&D). Cells were propagated in adherent monolayer cultures from passage (P) 0 until P3, without addition of differentiation factors. Astrocyte differentiation was initiated using a panel of differentiation factors, previously associated with astrocytic differentiation [23–28] (Supplementary Table 2). The panel was applied one day after plating of P3-astrocytes and maintained for 4 days. The cyclic adenosine monophosphate (cAMP) activator forskolin was used for downstream experiments [29].

### Primary culture validation using subventricular zone stem cells

The subventricular zone (SVZ) of female adult C57BL/6j mice (Charles River, Germany), age three to five months, was isolated in line with Johansson et al. [30]. SVZs were mechanically and enzymatically dissociated (Supplementary Table 3). Deoxyribonuclease I was added to minimize the presence of free-floating DNA fragments. The enzymatic reaction was stopped by adding 1% Bovine Albumin Fraction V diluted in Leibovitz's L-15 Medium + GlutaMAX™ Supplement. Dissociated cells were centrifuged (280 rcf, 5 min), and cells were washed in Leibovitz's L-15 Medium + GlutaMAX™ Supplement. Cells were resuspended in expansion medium (Supplementary Table 3). SVZ propagation into neural spheres was conducted as previously described [31]. EGF (20 ng/mL, Sigma-Aldrich, Supplementary Table 3) and FGFb (5 ng/mL, R&D Systems, Supplementary Table 3) were added every second day. All cells underwent two passages. The proliferating neural stem/progenitor cells formed spheres and underwent the first passage after 5 to 7 days incubation, depending on sphere size. Following passage, cells were seeded in expansion medium at approximately 300 * 10^3^ cells/plate. P2 followed after 7 days. Dissociated cells were plated at a density of 35 * 10^3^ cells/well in 96-well plates coated with poly-D-lysine hydrobromide (20 ng/ml, Sigma-Aldrich, Supplementary Table 3) in astrocyte medium (Supplementary Table 2) supplemented with EGF (20 ng/ml, Supplementary Table 2) and FGFb (25 ng/ml, Supplementary Table 2) (R&D Systems, Supplementary Table 2). Growth factor-supplemented medium was changed every second day. On the seventh and ninth day following plating, differentiation was prompted by only supplementation with forskolin (FSK) [29] (Sigma-Aldrich) at a concentration of 10 µM. On day 12 following plating, cells were fixed or exposed to other downstream assays.

### ES- and SVZ-astrocyte functional assessment using neuroinflammatory stimulation

The astrocytic neuroinflammatory response seen in vitro [17, 19, 24] and in vivo [32] following stimulation was assessed using a combination of interleukin- (IL-) 1α (3 ng/ml, Sigma-Aldrich, Supplementary Table 4) and tumor necrosis factor- (TNF-) α (30 ng/ml, Cell Signaling Technology, Supplementary Table 4) as these have recently been shown to confer astrocyte-activation [17]. Cells were fixed at 2 or 24 h following stimulation, using 4% methanol-free paraformaldehyde (PFA, Thermo Fisher Scientific).

### Co-culture system between ES-astrocytes and ES-motor neurons

For co-cultures of ES-astrocytes and ES-motor neurons, P3 ES-astrocytes were stimulated with first FSK [29] for 5 days, followed by a neuroinflammatory mediator for 24 h. IL-1α (3 ng/ml, Sigma-Aldrich) and TNF-α (30 ng/ml, Cell Signaling Technology) were used as positive controls. Neuroinflammatory proteins recently demonstrated to be enriched in CSF following human TBI [33] were also used and included (Supplementary Table 4): complement component 1Q (C1Q) (400 ng/ml, MyBioSource), complement component 5 (C5) (500 ng/ml, EMD Millipore); IL1-β (5 ng/ml, R&D), IL-6 (10 ng/ml, R&D), and transforming growth factor- (TGF-) β2 (5 ng/ml, R&D). Protein concentrations were derived from Refs. [34–37].

For co-cultures, ES-motor neuron homogeneity was ensured using fluorescence-activated cell sorting with gating optimized for eGFP + . Two co-culture designs were applied. In the first, ES-motor neurons (density 15 kcells/well) were plated on top of ES-astrocytes (15–35 * 10^3^ cells/well) in 96-well plates. ES-astrocyte medium was not omitted before this. ES-motor neuron dissociation was conducted the same day as sorting and plating. In the second design, conditioned medium from the ES-astrocytes (plated in 6-well plates at density 0.99 *10^6^ cells/well) was transferred to an ES-motor neuron monolayer (~ 10 * 10^3^ cells/well in a 96 well-plate). Here, ES-motor neurons were dissociated the same day as the ES-astrocytes were exposed to the neuroinflammatory substances. Conditioned media transfer was conducted the subsequent day. Both designs thus inferred a small risk for residual synthetic cytokines/neuroinflammatory mediators within the cell-culture medium at the time of ES-motor neuron plating, but this is likely neglectable as demonstrated by the very short half-lives of these substances (Supplementary Table 4). Co-cultures were fixed 24 h following co-culture initiation.

### Immunocytochemistry

Cells fixed with 4% PFA were blocked in blocking buffer (Supplementary Table 5) for 1 h and then incubated with primary antibodies (Supplementary Table 5) at + 4° C overnight. Following washing buffer (Supplementary Table 5) rinse, cells were incubated with secondary antibodies (Supplementary Table 5) diluted at 1:500 for 1 h at room temperature. Following additional washing buffer rinse, cells were incubated for 15 min in 4’,6-diamidino-2-phenylindole dihydrochloride (DAPI, Invitrogen) diluted 1:25000. Cells were mounted in 1X PBS (Thermo Fisher Scientific) for imaging following final rinsing steps.

### Image acquisition and cell quantification

A confocal microscope (LSM 880 with Airyscan, Zeiss) was used for image acquisition. Images were obtained using the autofocus fluorescence mode for 96-well plate layout, with automatic acquisition of up to 5 × 5 tiles/well. For sparse amounts of cells, pre-determined locations in each well were optimized for DAPI expression, followed by “scan mode” image collection.

Image quantification was conducted using MetaMorph® (Molecular Devices). Images were preprocessed into 16 bit TIFF files, separated by channels and tiles through ZEN blue (Zeiss), and renamed into a consecutive numerical sequence using the IrfanView software [38]. Images of poor quality were excluded prior to analysis. Images were quantified with regard to total number of cells, cells positive for different stainings, and cells positive for multiple combinations of stainings using a custom-made “journal” (MetaMorph®’s annotation for user-interface based scripts) for the Multiwavelength cell scoring and Neurite Outgrowth tools in MetaMorph®.

### Flow cytometry

We adapted a protocol from Berglund and colleagues [39]. For the experiments we used day 6 EBs (*n* = 4 biological replicates; each biological replicate containing > 1 * 10^6^ cells), thawed at day 5. We used thawed day 3 EBs as negative controls as these had not yet commenced to express eGFP. Cells were dissociated using 0.05% trypsin (Gibco) into single-cell suspension diluted into sterile-filtered flow cytometry-buffer (Supplementary Table 6). Cells were stained using a live/dead dye (Invitrogen, Supplementary Table 6) at 1:500, followed by PFA (2–4%) fixation. Using a permeabilization kit (eBioscience™ Foxp3/Transcription factor staining buffer set, Thermo Fisher Scientific, Supplementary Table 6), cell permeabilization, followed by staining with directly-conjugated Ki67 (BD Bioscience) was conducted. Cell acquisition was analyzed using a BD LSR Fortessa flow cytometer (BD Bioscience). Data was analyzed using the Kaluza software (Beckman Coulter).

### Fluorescence-activated cell sorting

For FACS experiments, we used a SH800 Cell Sorter (Sony Biotechnology), and the Cell Sorter Software (version 2.1). The sorting chip nozzle diameter was 130 μm. Lasers were set to λ-488nm. Sample pressure was kept at 6–10 to maintain an event-rate per second ≤ 3,000, in line with previous FACS experiments on stem-cell derived (motor) neurons [40, 41]. Cells were prepared as a single-cell suspension in FACS buffer **(**Supplementary Table 6**)**, filtered through a 40 μm cell-strainer (VWR). For isolation of the eGFP + fraction of dissociated EBs we used the “purity” mode and “two-way tube sorting” for cell sorting and collection. Cells (15 kcells/well) were sorted into fresh motor neuron medium (Supplementary Table 1). For separation of co-cultivated ES- astrocytes and eGFP + ES-motor neurons we sorted cells into a 96-well plate (~ 200 cells/well), filled with 4 °C lysis buffer, and the sorting setting “single cell mode”.

### Library preparation and RNA-sequencing

We conducted library preparation for cytosolic polyA + RNA using FACS-sorted cells as was recently described for parallelized direct nuclear tagmentation and RNA-sequencing [42]. We refer readers to the protocol by Zachariadis et al. [43] for reagents and procedures. In brief, we lysed ~ 200 cells/sample (n = 4 biological replicates per treatment group) in a lysis buffer containing dNTP and oligo-dT primers. PolyA + RNA was converted to cDNA using a reverse transcriptase reaction, including a template switch mechanism at the 5’ end of the RNA transcript [44], originally employed in the Smart-Seq [45] and Smart-Seq2 [46] protocols to obtain full-length cDNA strands for amplification. For cDNA fragmentation and barcoding, we employed the Tn5-based tagmentation procedure [47], as recommended by Picelli and co-workers [46]. Albeit originally developed for single-cell RNA-sequencing, this methodology is equally applicable for bulk RNA in small numbers [46]. We used Illumina-compatible barcodes (8 base pairs, dual-index). Pooled libraries were sequenced on Illumina NextSeq 550 using paired-end sequencing with 91 cycles. Samples were de-multiplexed and reads were mapped at the sequencing facility using STAR [48], run from a Linux server. Raw data and de-multiplexed raw counts have been deposited in NCBI’s Gene Expression Omnibus [49] and are accessible through GEO Series accession number GSE213804 (https://www.ncbi.nlm.nih.gov/geo/query/acc.cgi?acc=GSE213804).

Bulk RNA sequencing experiments where SVZ cells were utilized as negative controls before initiation of differentiation were undertaken and are described in Supplementary Methods.

### Statistical analysis

We used R (version 4.1.1) [50], through the graphical user interface RStudio® (version 1.4.1717). General statistical operations were carried out using the RColorBrewer [51], cowplot [52], gridExtra [53], and tidyverse [54] packages. Technical replicate medians/means were used to calculate mean biological replicate raw data value, from which summary statistics were derived. One biological replicate was defined as one unique vial from a defined passage number for ES cells, and one unique experimental animal for SVZ cells. Two- or multiple-group comparisons were preceded by the Shapiro Wilk test for normality assessment and the Levene test for variance homogeneity using the car package in R [55]. For two-group comparisons fulfilling these criteria, the Student’s t-test was employed. For multiple-group comparisons fulfilling these criteria, a one-way ANOVA followed by Tukey post-hoc testing was conducted. For two-group comparisons not fulfilling these criteria, the Wilcoxon rank sum test was conducted. For multiple-group comparisons not fulfilling these criteria, the Kruskal Wallis test, followed by the Dunn post hoc test was conducted using the R packages FSA [56] and onewaytests [57]. In parallel, linear regression analysis was applied as the focus for the majority of assessments were primarily differences between treatment groups versus control treatment and less often other between-treatment group differences. Independent of analysis, a *p* value (or if multiple comparison, the multiple-comparison adjusted *p* value) ≤ 0.05 was considered significant.

For RNA-sequencing data analysis, we used Ensembl-IDs (*Mus musculus*) for gene annotation through the Bioconductor package biomaRt [58, 59]. Raw read counts and sample metadata were converted to a DESeqDataSet using the Bioconductor package DESeq2 [60, 61]. Explorative data analysis were conducted on prefiltered data (read counts > 1) that were subjected to a variance stabilizing transformation using the rlog() function. Distance matrices derivation and cluster analyses were conducted using the Bioconductor packages DESeq2, M3C, and PoiClaClu [60–63]. For differential gene expression analysis, we employed the DESeq() function of DESeq2 [60, 61] with the false discovery rate set to 10%. A significant differentially expressed gene was defined to exhibit log_2_FC >|1| (or as stipulated in-text), and a FDR-adjusted *p*-value ≤ 0.05. For volcano plots, we used in parallel the non-shrunken and shrunken log_2_ fold changes, together with the adaptive Student’s t prior shrinkage estimator, available through the Bioconductor package apeglm [64]. For gene set enrichment analyses and pathway analyses, we did not use shrunken log_2_ fold changes. Gene set enrichment analysis [65] were conducted using the Bioconductor package fgsea [66], for the gene sets Hallmark [67], Reactome [68], Gene Ontology (GO) [69], and the Kyoto Encyclopedia of Genes and Genomes [70]. Gene sets were acquired through the Molecular Signatures Database available from the Broad Institute [71] through the Bioconductor package msigdbr [72]. GO annotations were accessed using the Bioconductor package GO.db [73]. All custom-written code is available upon request to the corresponding author.

### Data availability

The datasets used and/or analyzed within the current study are available from the corresponding author on reasonable request. For RNA sequencing data, raw data and de-multiplexed raw counts have been deposited in NCBI’s Gene Expression Omnibus [49] and are accessible through GEO Series accession number GSE213804.

(https://www.ncbi.nlm.nih.gov/geo/query/acc.cgi?acc=GSE213804) and GEO Series accession number GSE232232 (https://www.ncbi.nlm.nih.gov/geo/query/acc.cgi?acc=GSE232232).

## Results

### Ventral brainstem and rostroventral spinal glial progenitors can be differentiated into ES-astrocytes

**Fig. 1:**
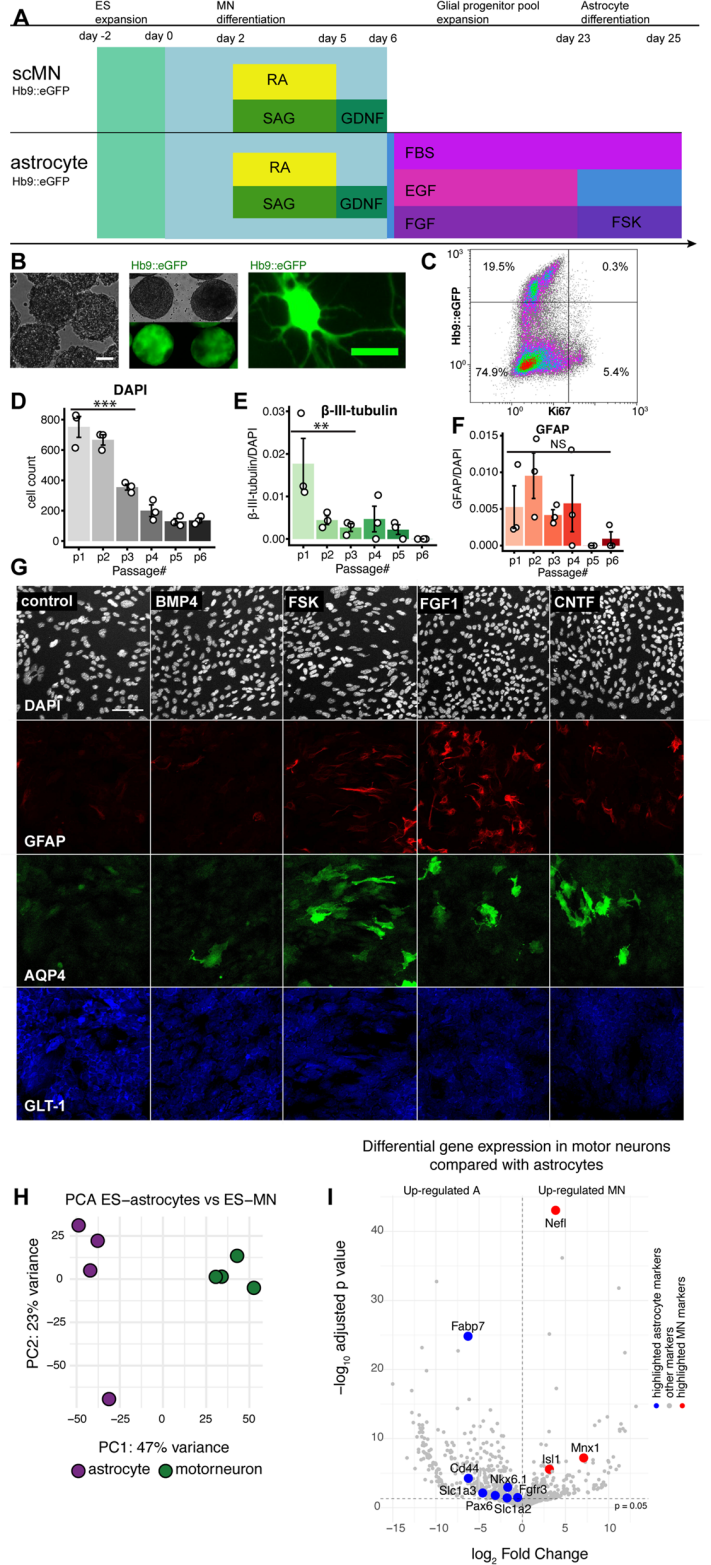
Differentiation of embryonic stem cells into brainstem/spinal motor neurons and astrocytes. By mimicking neurogenesis in vivo, brainstem/spinal motor neurons followed by astrocyte-like cells from the same regional niche were generated (**A**). Motor neuron genesis was monitored by Hb9∷eGFP expression. Scale bars (left to right): 50 μm, 50 μm, 25 μm. **B** At day 8 following plating cultures comprised post-mitotic motor neurons (Hb9 + , Ki67-) and interneurons (Hb9-, Ki67-) as well as a small pool of glial progenitors (Hb9-, Ki67 +). **C** The glial progenitors were further expanded across multiple passages (P), upon which a sharp decrease in proliferation could be observed at P3 (**D**), as well as low β-III-tubulin expression, indicating a successively homogenous glial cell pool (**E**). GFAP expression was constant across passages (**F**). Following addition of the cAMP activator forskolin, or other differentiation factors, we could verify protein expression of mature astrocytic markers. Scale bar: 100 μm. **G** PolyA + bulk-RNA sequencing verified distinct genomic profiles of the ES-derived astrocyte-like cells and ES-derived motor neurons (**H**), of which canonical genes were upregulated in the respective cell types (**I**). Significance level: NS, non-significant; *, *p* ≤ 0.05; **, *p* ≤ 0.01. Abbreviations: cAMP, cyclic adenosine monophosphate; ES, embryonic stem cell; FBS, fetal bovine serum; GFAP, glial fibrillary acidic protein; GLT-1, glutamate transporter 1; Hb9, homeobox Hb9; P, passage; sc, spinal cord/brainstem

The developing CNS is patterned through a complex interplay between spatial and temporal cues [74], initiated by neurogenesis [75]. In the brainstem and rostroventral spinal cord, motor neuron genesis is dependent on retinoic acid and Sonic hedgehog [22], and exposure to these factors in vitro generates postmitotic motor neurons from ES-cells [22]. Employing this strategy **(**Fig. 1A**)**, we could generate Hb9 + motor neurons, using a transgenic pluripotent mouse ES-cell line that expresses eGFP under the Hb9 promoter **(**Fig. 1B**)**. As expected, our cultures were heterogeneous, with in mean 19% (SD 2%) of cells expressing Hb9 + with Ki67-, indicative of postmitotic motor neurons **(**Fig. 1C**)**. This yield is in line with what has been previously reported [76]. The remainder of cells comprised interneurons (Hb9-, Ki67-), and in mean 5% (SD 1.4%) glial progenitors (Hb9-, Ki67 +).

Neurogenesis in vivo is succeeded by a gliogenic switch, causing the emergence of astrocytes and oligodendrocytes [75]. We thus assumed that the glial progenitors could be further differentiated into astrocyte-like cells and maintained cultures of ES-derived glial progenitors across multiple passages in FBS-containing medium with proliferative growth factors **(**Fig. 1A**)**. At P3 there was a distinct decrease in number of cells compared with P1, indicative of a diminished self-renewing potential **(**Fig. 1D, *p* < 0.001**)**. At the same passage, there was a significant decrease in the neuronal marker β-III-tubulin (Fig. 1E, *p* = 0.003), indicative of serum-mediated neuronal toxicity [77]. Of note, the mature astrocyte marker glial fibrillary acidic protein (GFAP) [78] was not significantly altered across passages (Fig. 1F, *p* = NS). In order to increment ES-astrocyte maturation at P3, we withdrew proliferative growth factors and supplemented the cultures with bone-morphogenetic protein 4 (BMP-4), ciliary neurotrophic factor (CNTF), acidic fibroblast growth factor (FGF1), or FSK, as these have been implicated in astrocyte differentiation [23, 25–28, 79]. We subsequently assessed the expression of the mature astrocytic markers GFAP, aquaporin-4 (AQP4), and glutamate transporter 1 (GLT-1) [80, 81]. We were predominantly interested in AQP4 + astrocytes as these are important in the neurovascular unit [82]. Notably, all treatments, including FBS-enriched medium alone (control), were superior to BMP4 **(**Fig. 1G**)**. There were no significant differences between CNTF, FGF1, and FSK supplementation with regard to AQP4-expression. GFAP was more enriched in FGF1-treated compared with FSK-treated astrocyte-like cells (*p* = 0.049). No significant differences were detected in GLT1-expression. Based on this, we proceeded with FSK as differentiation factor, given its recent implications in AQP4-mediated CNS edema [29].

We also compared the transcriptomes of the ES-derived cell types using a co-culture system of ES-motor neurons together with FSK-treated ES-astrocytes. Samples clustered depending on cell type origin, with cell type heterogeneity demonstrating 47% of transcriptomic variance **(**Fig. 1H**)**. Rostro-caudal positional identity genes (*Hox*) were non-significant between the two cell-types, indicative of a common origin. Among these, all *n* = 4 of Hox 4 paralogs were found (*Hoxa4-Hoxd4*), thus delineating the cells’ brainstem/rostral spinal cord origin along the rostro-caudal axis [83–85]. Gene expression was similar with regard to genes encoding Slit1 (*Slit1)* and Reelin (*Reln*) [86]. The ES-astrocyte positional identity in the ventral domain of the brainstem/rostral spinal cord could be verified through the ES-astrocytic upregulation of *Pax6*, and even more importantly *Nkx6.1* [85, 86], as the latter has been shown to be brainstem astrocyte-specific [85]. In line with this, control data of stem cells from the subventricular zone did not express *Nkx6.1*. As expected, a multitude of additional genes were differentially expressed between the two cell types **(**Fig. 1I**)**. Neuronal cell identity was confirmed through the upregulation of *Nefl*, encoding the neuronal protein neurofilament light [87, 88] (log_2_ fold change 3.9, p_adjusted_ < 0.001). ES-motor neuron identity could be confirmed through the upregulation of *Mnx1*, encoding Hb9 [89] (log_2_ fold change 7, p_adjusted_ < 0.001), but also other motor neuron-specific markers such as *Isl1* [90]. We compared the differentially expressed genes with the pivotal microarray data of astrocyte-enriched genes by Cahoy and colleagues [91]. We found upregulation of numerous canonical astrocytic genes, such as the early expressed *Slc1a3* (encoding glutamate aspartate transporter [GLAST]), *Fabp7*, and *Fgfr3* [80]. We also found enrichment of mature astrocytic genes, among else *Slc1a2* (encoding GLT-1) [81], and *Cd44* [79]. We thus generated ES-astrocytes with positional identity in the ventral part of the caudal brainstem, that were highly astrocyte-like, with regard to both transcriptomic and surface antigenic features. We next sought to characterize these cells in the context of neuroinflammation.

### ES-astrocytes respond to neuroinflammation by activating a c-Jun N-terminal kinase-dependent pathway

**Fig. 2:**
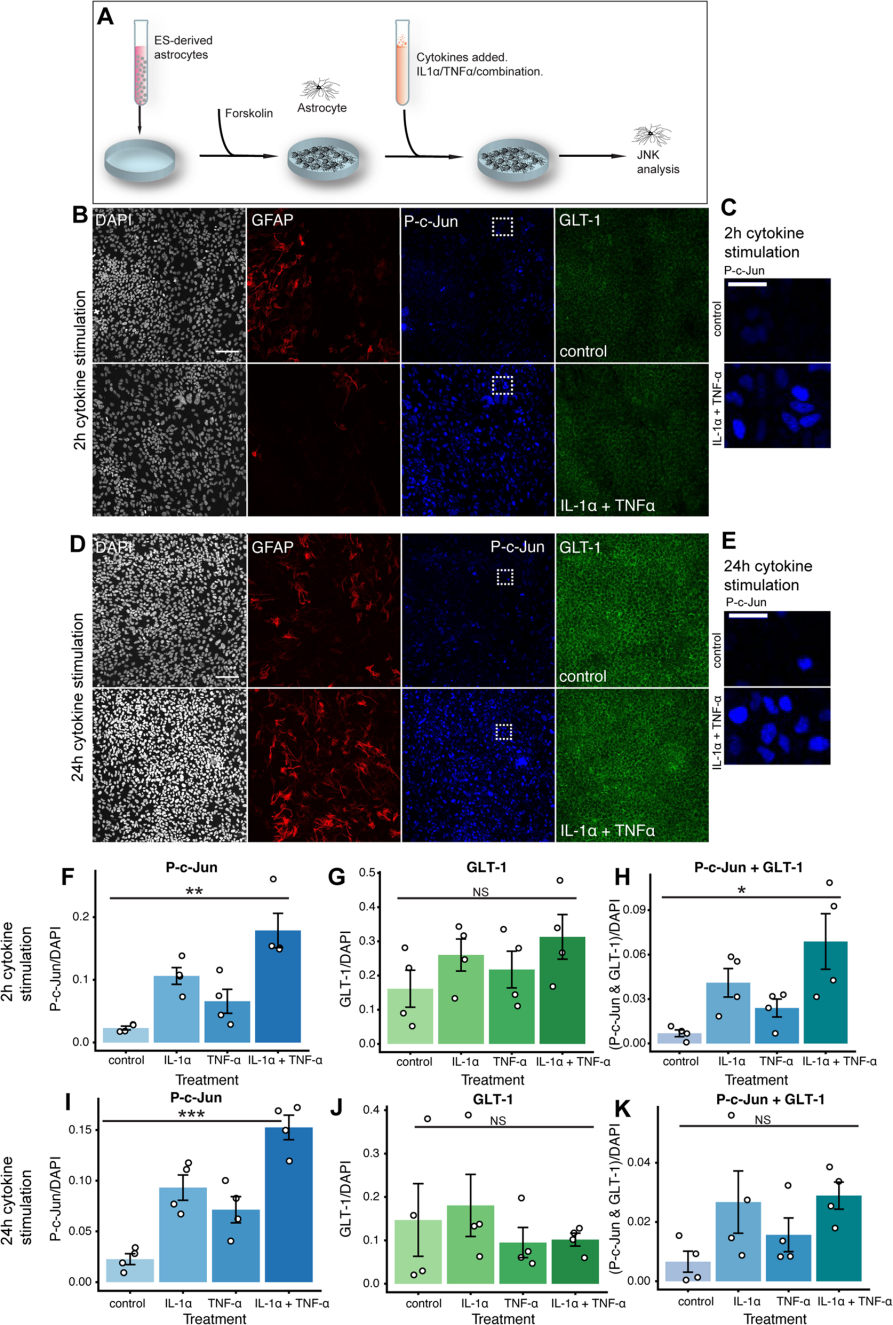
Embryonic stem cell-derived astrocytes undergo activation in response to a neuroinflammatory stimulus. We used the c-Jun N terminal kinase (JNK) pathway, a three-tiered mitogen-activated protein kinase pathway (MAPK) [95], to evaluate neuroinflammatory stimulation of ES-derived astrocyte-like cells by assessing the phosphorylation of c-Jun at either the Serine site 63 or 73. We exposed our cells to cytokines and then evaluated the JNK pathway (**A**) after both 2 h following cytokine stimulation (**B**, **C**), and 24 h after cytokine stimulation (**D**, **E**). At 2 h, P–c-Jun was increased in treated groups (**F**), while GLT-1 expression was similar across treatment groups (**G**), indicative that the maturity state of the cells was not altered, and that the astrocyte-like cells were afflicted by this stimulus (**H**). Congruent findings were seen after 24 h of cytokine stimulation (**I**-**K**). Significance level: NS, non-significant; *, *p* ≤ 0.05; **, *p* ≤ 0.01, ***, *p* < 0.001. Abbreviations: ES, embryonic stem cells; GLT-1, glutamate transporter 1; IL-1α, interleukin 1α; JNK, c-Jun N-terminal kinase; MAPK, mitogen activated protein kinase; P–c-Jun, phosphorylated c-Jun; Ser, serine; TNF-α, tumor necrosis factor α. Scale bars: **B**, **D**: 100 μm; C, E: 25 μm

An important role of astrocytes in vivo is their injury response, denoted *reactive astrogliosis* [92]. It was recently shown that a neurotoxic astrogliotic subtype was elicited via the cytokines IL-1α, TNF-α, and C1Q [17]. IL-1α and TNF-α both activate the c-Jun N-terminal kinase (JNK) pathway [93–95], a three-tiered mitogen-activated protein kinase pathway (MAPK) [95] ultimately converging on the MAPK protein JNK. This leads to the phosphorylation of c-Jun at either the Serine site 63 or 73 (P–c-Jun). P–c-Jun forms a homo- or heterodimer, denoted AP-1, that initiates a plethora of downstream transcriptional activities [95–97]. The JNK-AP-1 pathway, albeit versatile in function, is broadly implicated in glial cells in response to inflammation [98], and specifically in astrogliosis [99]. C1Q, in contrast, has been hypothesized to signal through the Multiple EGF-like domains 10 receptor on astrocytes [100]. In non-mammals, the equivalent receptor is known as Draper. As Draper is a downstream target for AP-1 [101] rather than an upstream inducer of the JNK pathway, we decided to assess IL-1α and TNF-α-induced astrocyte activation together while omitting C1Q-induced astrocyte activation. We thus induced astrogliosis in our ES-derived astrocyte-like cells using IL-1α together with TNF-α and evaluated P–c-Jun at serine site 63 and/or 73 **(**Fig. 2A**)**. Already at 2 h following cytokine stimulation, P–c-Jun was increased in a subset of IL-1α and TNF-α treated cells compared with unstimulated cultures (Fig. 2B, C, F, *p* = 0.003). The mature astrocytic marker GLT-1 was stable across treatment groups (Fig. 2G, *p* = NS). When examining co-localization between GLT-1 and P–c-Jun, the treatment effect was still significant (Fig. 2H, *p* = 0.023), indicating that the treatment effect was not exclusive to residual glial progenitor cells. Interestingly, the cytokine-induced JNK-increase was sustained over time, and after 24 h of cytokine-exposure, P–c-Jun was still increased in cytokine-stimulated cells compared with control (Fig. 2D-E, I, *p* < 0.001), while GLT-1 was stable across treatment groups (Fig. 2J, *p* = NS). Co-localization between P–c-Jun and GLT-1 was less clear in this group (Fig. 2K, *p* = NS). Of note, GFAP was not a good marker of astrogliosis at either time-point (Fig. 2B, D, Supplementary Fig. 1A-B). Taken together, this indicates that the mouse ES-astrocytes shared similarities with *bona fide* astrocytes with regard to transcriptome, surface antigens, and hallmark functional features [78–81, 86, 91, 92].

### Subventricular zone-astrocytes respond to neuroinflammation similarly to ES-astrocytes

We further validated the stem-cell approach by applying the ES-astrocyte culture protocol and assays on primary culture mouse SVZ-astrocytes (Supplementary Fig. 2A). We chose this cell type rather than other primary astrocyte subtypes since SVZ-astrocytes are neural stem cells in the adult animal that both exhibit glial features [102], and respond to inflammatory stimulus [103]. Similarly to the ES-astrocytes, SVZ-astrocytes exhibited a high extent of canonical astrocytic markers such as Nuclear factor 1A [104], vimentin [105], AQP4 [80], GLT-1 [81], and GFAP [78] (Supplementary Fig. 2B-D). Further, SVZ-astrocytes also responded by increasing P–c-Jun within the JNK pathway following neuroinflammatory stimulus (Supplementary Fig. 2E-F, *p* = 0.006). Importantly, the JNK pathway activation could be inferred to SVZ-astrocytes specifically by examining colocalization of P–c-Jun with canonical astrocytic markers such as GLT-1 (Supplementary Fig. 2G, *p* = 0.02). Taken together, ES-astrocytes shared similarities with SVZ-astrocytes. We deemed ES-astrocytes suitable for modeling astrobiological phenomena in vitro.

### Reactive ES-astrocytes may exert a contact-dependent neurotoxic effect on ES-motor neurons

**Fig. 3:**
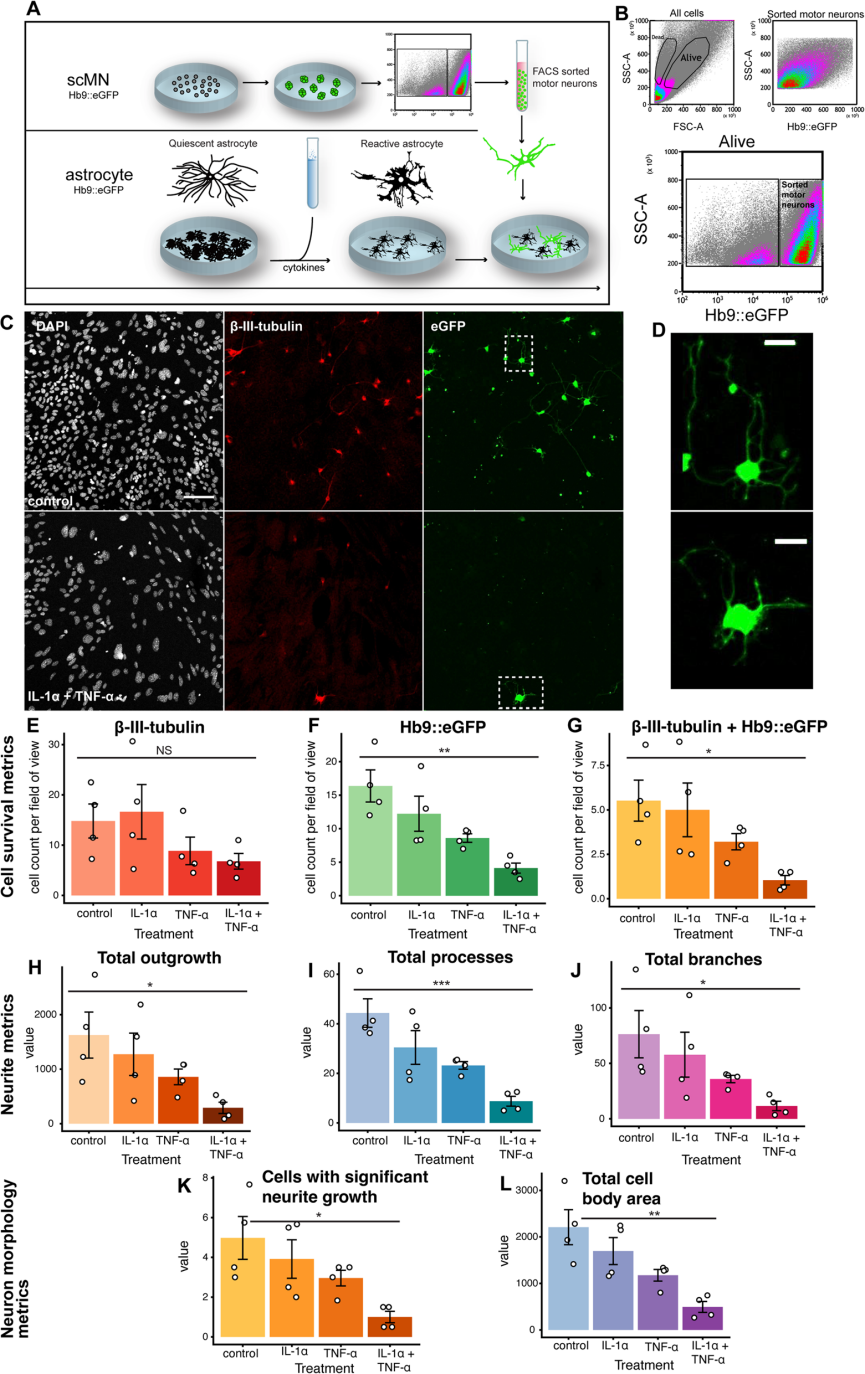
Co-culture system of astrocyte-mediated neurotoxicity. We cultured brainstem/spinal cord motor neurons and astrocytes from the same region in parallel (**A**). We plated FACS sorted motor neurons (**B**) on top of cytokine stimulated reactive astrocytes (**A**). Following co-culture, motor neuron survival decreased distinctly when motor neurons were cultured on top of reactive compared with quiescent astrocytes (**C**, **D**). Whereas there was a trend for decreased overall neuronal survival (**E**), motor neurons significantly decreased (**F**-**G**). Moreover, among surviving and dying motor neurons, both neurite (**H**-**J**), and neuron morphology (**K**-**L**), altered. Significance level: NS, non-significant; *, *p* ≤ 0.05; **, *p* ≤ 0.01, ***, *p* < 0.001. Abbreviations: DAPI, 4’,6-diamidino-2-phenylindole dihydrochloride; FACS, fluorescence activated cell sorting; GFP, green fluorescent protein; Hb9, homeobox Hb9; IL-1α, interleukin 1α; MN, motor neuron; sc, spinal cord/brainstem; SSC-A, side scatter; TNF-α, tumor necrosis factor α. Scale bars: **C**, 100 μm, **D**, 25 μm

The neurotoxic effect described for forebrain astrocytes stimulated with IL-1α, TNF-α, and C1Q [17] has also been described in hiPSC-astrocytes with a spinal identity [19]. In both studies, the astrocyte-mediated neurotoxic effect was evaluated using cortical neurons. Whether the neurotoxic effect of reactive astrocytes also apply to motor neurons in the brainstem/spinal cord is unclear. We tested this hypothesis by exposing ES-astrocytes to IL-1α and TNF-α for 24 h. Next, we plated FACS-sorted ES-motor neurons on top **(**Fig. 3A, B**)**. Interestingly, ES-motor neurons but not ES-interneurons died following co-cultivation (Fig. 3C,E-G, *p* = NS for β-III-tubulin, *p* = 0.0025 for Hb9∷eGFP, and *p* = 0.030 for β-III-tubulin + Hb9∷eGFP). Moreover, we found important differences in ES-motor neuron neurite metrics (Fig. 3D,H-L). ES-motor neurons had shorter neurites (Fig. 3H, *p* = 0.037), fewer neurites (Fig. 3I, *p* < 0.002), and fewer neurite branches (Fig. 3J, *p* = 0.042). Notably, fewer ES-motor neurons fulfilled viability criteria as measured per significant neurite growth (Fig. 3K, *p* = 0.015). ES-motor neurons also had a smaller cell body area (Fig. 3L, *p* = 0.002). In order to examine whether this effect was contact-dependent or paracrine in nature, we cultured ES-astrocytes and ES-motor neurons separately. After 24 h of ES-astrocyte cytokine exposure, conditioned media was transferred to the ES-motor neurons (Supplementary Fig. 3A). In general, very few cells survived this procedure, independent of treatment (Supplementary Fig. 3B). This indicates that the shear stress inferred from the FACS on the ES-motor neurons is severe, and that ES-motor neurons following FACS need trophic support from ES-astrocytes. Onwards, we therefore pursued contact-dependent assays. Importantly, we can conclude that ventral brainstem and/or rostroventral spinal ES-astrocytes have the potential to undergo a neurotoxic switch, causing decreased survival of neurons with the same regional identity.

### Trauma-relevant neuroinflammatory stimuli induce a neurotoxic potential in ES-astrocytes

**Fig. 4:**
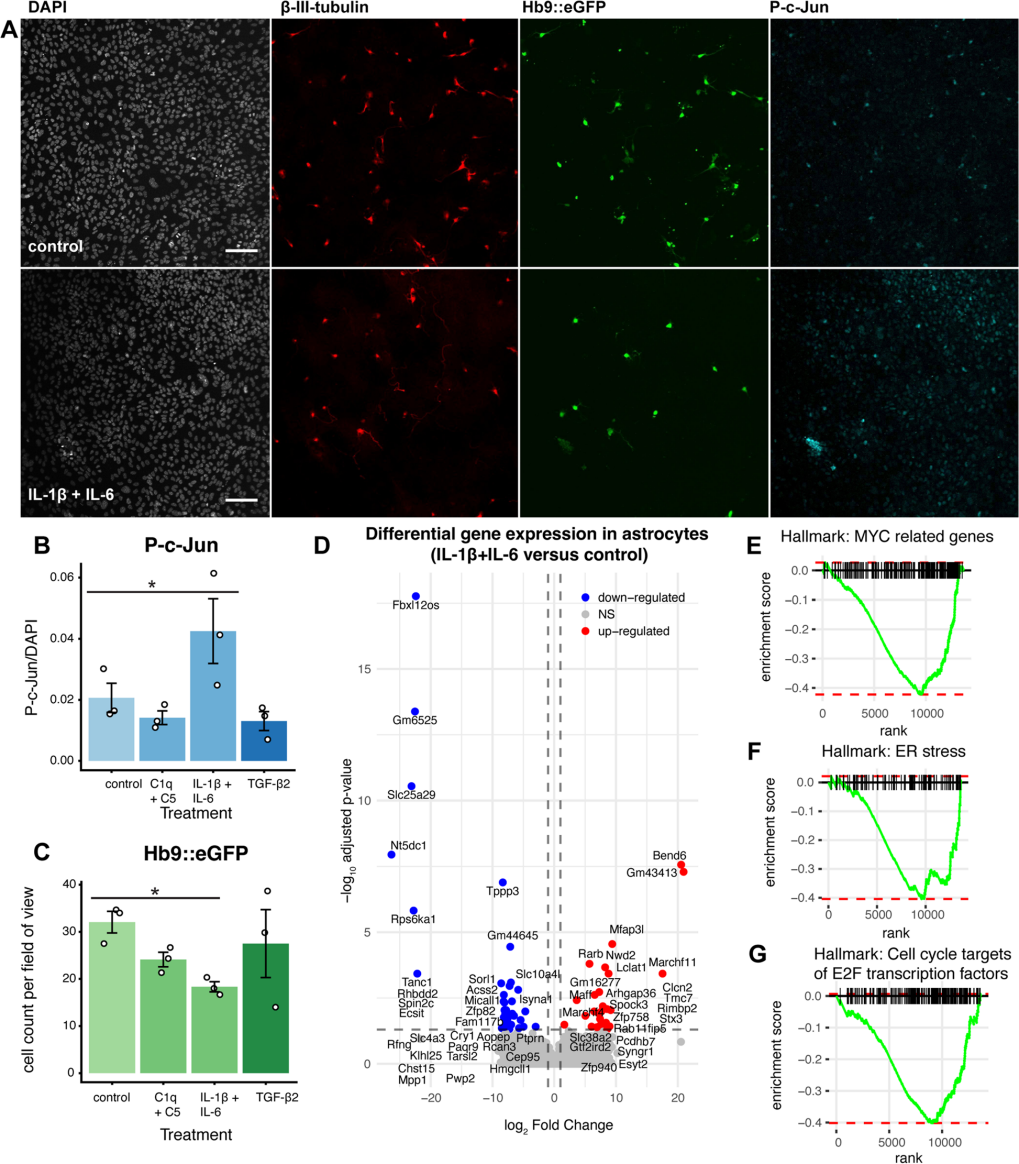
Translational relevance of the neurotoxic mechanism and hypothesis-generating mechanistic explanations. Contact-dependent experiments were pursued by co-culturing reactive ES-derived astrocytes stimulated with a panel of clinically relevant neuroinflammatory factors together with motor neurons (**A**). Following astrogliosis induced by IL-1β and IL-6, we saw the largest P–c-Jun upregulation (**B**), and a concordant astrocyte-mediated neurotoxic effect on motor neurons (**C**). Hypothesis-generating polyA + bulk-RNA-sequencing of FACS-sorted reactive astrocytes (**D**) demonstrated some tentative pathways possibly implicated in this (**E**–**G**), of which some are also implicated in the c-jun N terminal kinase pathway [95] (**E**). Significance level: NS, non-significant; *, *p* ≤ 0.05. Abbreviations: DAPI, 4’,6-diamidino-2-phenylindole dihydrochloride; ER, endoplasmic reticulum; eGFP, enhanced green fluorescent protein; Hb9, homeobox Hb9; IL-1α; IL-1β, interleukin 1β; IL-6, interleukin 6; P–c-Jun, phosphorylated c-Jun; TNF-α, tumor necrosis factor α. Scale bars: 100 μm

We recently described proteins associated with blood–brain barrier integrity, long-term functional outcome, and neuroinflammation in the cerebrospinal fluid and blood proteome following severe human TBI [33]. From these, we selected the innate immunity proteins complement components C1Q, C5, IL-1β, IL-6, and TGF-β2 [33]. We used these as inducing cytokines in the contact-dependent co-culture system **(**Fig. 4A, Supplementary Fig. 4) to study if these proteins were implicated in ES-astrocyte-mediated ES-motor neuron death. The cytokines IL-1β and IL-6 are core neuroinflammatory mediators following both experimental and clinical TBI [106] and as some authors suggest they are inter-dependent [107], we assessed them jointly in our set-up. In a similar line of reasoning, we also combined the complement components C1q and C5 (Fig. 4B, C, Supplementary Fig. 4) together. Among all neuroinflammatory groups, JNK pathway upregulation measured using P–c-Jun was highest in IL-1β and IL-6 treated cells (Fig. 4B, *p*=  0.035). Among these cells, the ES-derived astrocyte-like cells also conferred neurotoxicity (Fig. 4A, C *p*= 0.038). Importantly, this demonstrates that our ES-derived co-culture system of brainstem astrocyte-like cells and motor neurons can be used to model astrocyte-mediated neurotoxic effects using clinically relevant neuroinflammatory stimuli.

The data presented implicates a JNK-dependent pathway as a downstream mediator for astrogliosis. However, other pathways could also be implicated in mediation of the ES-astrocyte-induced neurotoxic effect. To explore this, we undertook hypothesis-generating polyA + RNA sequencing of ES-astrocytes and ES-motor neurons following co-culture. We explored expression differences between IL-1β and IL-6 treated ES- astrocytes compared with control-treated ES-astrocytes (*n* = 4 biological replicates per treatment group). Among 64 differentially expressed genes **(**Fig. 4D**)**, tentative pathways of future interest for exploration pertained to MYC-regulated genes (normalized enrichment score [NES] -1.76, p_adjusted_ = 0.017), cell cycle mechanisms (NES-1.67, p_adjusted_ = 0.017), and endoplasmic reticulum-stress (NES -1.56, p_adjusted_ = 0.038) **(**Fig. 4E-G**)**. Within the leading edges of these respective gene sets, we found numerous genes recently implicated in a landmark study of astrocyte reactivity, among else *Mthfd2*, *Ccl2*, *Tubb2a*, *Cdkn1a, Gins1, Prdx4,* and *Ppia* [9], thus corroborating a neuroinflammation-induced astrogliosis. Moreover, leading edges also contained genes where the corresponding protein has been shown to be enriched following focal TBI, such as *Ranbp1*, *Hspa5, Prdx3, Vegfa*, and *LDHA* [33, 108]. Interestingly we also found genes that have been shown to be up-regulated on the protein-level following TAI, such as *Psma1*, and notably, upregulated in TAI compared with focal TBI (*Ywhaq*) [108].

## Discussion

We present an in vitro study of ventral brainstem/rostroventral spinal astrocytes and motor neurons derived from mouse embryonic stem cells. We show the resemblance between ES-astrocytes and mature mouse astrocytes with regard to transcriptome, protein expression, and functional features. Importantly, we show that ES-astrocytes following neuroinflammatory stimulation with TBI-relevant cytokines adopt a neurotoxic fate, thus possibly representing an additional secondary mechanism ensuing the primary axonal injury seen after CNS trauma.

### Regional identity is important in astrocyte and motor neuron co-culture studies

Stem-cell based model systems have become important translational research tools in neuroscience as access to human CNS cells is limited. We utilized ES-cells to differentiate ES-astrocytes. Although these cells were shown to share many hallmarks of *bona fide* astrocytes, there might be differences between the cell types unaccounted for as demonstrated for hiPSC-astrocytes that exhibit a relative immaturity [84]. Upon extended culture time, hiPSC-astrocytes undergo maturation similar to that seen in vivo [109], but this can generate unfeasibly long culture times in vitro. Over-expression of certain astrocytic transcriptions factors might solve this [110], as well as sorting methods targeted towards mature hiPSC-astrocytes [19]. As our cultures were not entirely homogenous and some cells thus likely retain immature features, we would have benefited from astrocytic sorting techniques. Yet, although stem cell-based model systems infer some confounding related to cell maturation, they entail a translational applicability otherwise difficult to achieve in neuroscience.

We chose to derive ES-astrocytes and ES-motor neurons that in vivo are situated in the ventral brainstem/rostroventral spinal cord. This is motivated in the context of traumatic CNS lesions, where the brainstem constitutes a particularly vulnerable region [10], but theoretically also across other CNS diseases. In this niche, motor neuron death has been linked to astrocytic malfunction, as shown in work on amyotrophic lateral sclerosis [20]. Presumably, interactions between astrocytes and motor neurons here could also be important across other pathologies. Recent work has implicated the cytokines we used (IL-1α, TNF-α, complement) in the context of forebrain [17] astrocyte-mediated neurotoxicity and spinal hiPSC [19] astrocyte-mediated neurotoxicity. Yet, no studies have compared brainstem/spinal (motor) neurons and brainstem/spinal astrocytes. This is however critical as astrocyte heterogeneity is vast, with profound regional implications [111, 112]. Astrocyte heterogeneity likely arises during embryonic stages, when astrocytes are positioned in a patterned fashion without signs of inter-compartmental movement [113]. This has functional implications under homeostasis as astrocytes support neurons with the same regional identity but *not* others [114]. This highlights the importance for astrocytic-neuronal co-cultures with the same regional identity, as neuronal responses otherwise may be confounded by regional mismatch. Reactive astrocytes also harness heterogeneity among them. Initial work demonstrated injury-specific genetic astrogliotic signatures [6]. More recent work utilizing one inductive stimuli followed by single-cell resolution examination of cortical astrocytes identify at least nine gene clusters with both regional and reactive identity [9]. These differences likely hold functional implications. It was e.g. shown that the astrocytic secretome of ventral spinal cord astrocytes is disparate from that of forebrain astrocytes following similar stimuli [18]. Our model system holds the benefit of portraying one stringent CNS niche. This strengthens the biological relevance of our findings, at the expense of limiting their extrapolation to other CNS regions.

### Astrocyte-mediated neurotoxicity might represent a CNS injury mechanism available for treatment

Following traumatic CNS lesions, numerous cellular injury mechanisms are prompted [115], among else (neuro)inflammation [116]. Here, we demonstrate that ES-astrocytes may adopt a neurotoxic phenotype, depicted through co-culture with ES-motor neurons. All astrocyte culture media contained FBS, well-known to prompt astrocytic gene alterations [117], and notably also astrocyte reactivity [118, 119]. Yet, the neurotoxic response seen can be inferred to the addition of the neuroinflammatory cytokines, due to identical culturing conditions across all treatment groups. This neuroinflammatory response was seen independent if the reactive ES-astrocytes were induced using the previously described “A1-inducing cytokines” [17, 19] or with the clinically relevant [33] neuroinflammatory factors IL-1β and IL-6. Previously, TGF-β has been shown to revert the neuroinflammatory response [17]. We did not find any such effect, possibly since FBS contains a high concentration of TGF-β at baseline [120, 121]. Taken together, ES-derived mouse astrocytes respond to neuroinflammatory stimulus of clinical relevance.

Even though we show a strong association between Jun activation and motor neuron death, we do not claim this to be a causal mechanism. Rather, Jun expression is used here as a non-specific biomarker for stress. As the inductive stimuli are inflammatory mediators, this stress is likely inflammatory, as shown by transcriptomic astrocytic alterations characteristic of astrogliosis. In contrast, motor neuron death here is an exclusive functional consequence of astrocytic inflammatory stimulation, where we demonstrate hypothesis-generating data for future work. We propose that astrocyte-induced neurotoxicity entails an additional cellular injury mechanism for neuronal death following e.g. trauma. Why astrocytes adopt a neurotoxic phenotype is beginning to unfold. Theoretically, an astrocyte could become “neurotoxic” due to loss-of-function, and consequently diminished homeostatic neuronal trophic support following CNS injury. This seems less likely as deletion of the A1-inducing cytokine genes in vivo results in improved neuronal survival [17]. Another explanation is that reactive astrocytes acquire a gain-of-function manifesting as neurotoxicity. Evidence for the latter has recently emerged, where astrocytes have been attributed to secrete lipids that confer the neurotoxic effect [122]. While we have no definitive mechanistic answers, we delineate a genetic signature in neurotoxic astrocytes with implications towards endoplasmic reticulum-stress and altered MYC pathway regulation. Albeit only correlative at this point, we find that several genes within the leading edges of these gene sets have been shown to be up-regulated at the protein level following TBI, including TAI [108], thus theoretically representing an avenue for further translational studies. Our findings must be expanded upon, preferably in a hiPSC system, as the mouse and human astrocytic genomes differ, especially with regard to inflammatory pathways [123].

Following CNS trauma and TAI, neurons are irretrievably lost. A tantalizing idea is to utilize stem cells differentiated into neurons as cell replacement. In order to succeed with this, the neurotoxic environment however likely needs to be modulated, either to halt neuronal loss or to optimize environmental survival-cues preceding cell replacement. Crude astrocyte obliteration is not feasible, as shown by in vivo work where astrocyte ablation both acutely or chronically was detrimental for neuronal survival in TBI [124], and for axonal regrowth in spinal cord injury [125]. These findings have been attributed to the astrogliotic scar, that encapsulates CNS lesions and limits the extent of inferred damage [7], thus demonstrating the duality of CNS neuroinflammation. A more complex idea might therefore be to selectively shift the astrocytic neuroinflammatory response. It has been proposed that TGF-β represents such a substance [17], and it has also been seen to be upregulated in pathways ensuing severe TBI in humans [33]. In our material, induction of astrocyte reactivity using TGF-β2 did not seem to infer any astrocyte-mediated neurotoxicity, possibly speaking in favor of this. Moreover, a recent study demonstrated that a mixture of three cytokines of which one was TGF-β1, improved outcome following TBI [126]. Paradoxically, the same study also demonstrated that IL-6 was beneficial for outcome [126]. This stands in contrast to our work, where a combination of IL-1β and IL-6 conferred an astrocyte-induced neurotoxic effect, and also previous work that has implicated IL-6 as a contributor to neurologic malfunction following mild TBI [127]. Whether these observed effects are due to a discrepant effect of IL-6 on peripheral and CNS immune cells remains to be elucidated. Astrocyte neuroinflammatory modulation might be a therapeutic avenue preceding cell-replacement of damaged CNS neurons. Future work must determine whether this strategy is feasible in the context of human cells and in vivo.

## Conclusion

Astrocyte-like cells derived from mouse embryonic stem cells hold the capacity to adopt a neurotoxic phenotype in the ventral brainstem/rostroventral spinal cord following induction with neuroinflammatory stimuli relevant in human severe TBI. This might represent an additional secondary mechanism ensuing the primary axonal injury seen after CNS trauma in the brainstem region.

## Supplementary Information

**Additional file 1: Supplementary Methods; Supplementary References.** **Supplementary Table 1. **Media constituents for motor neuron differentiation. **Supplementary Table 2. **Media constituents for astrocyte differentiation. **Supplementary Table 3. **Media constituents for primary SVZ cell culture. **Supplementary Table 4. **Neuroinflammatory factors used. **Supplementary Table 5. **Materials used for immunocytochemistry. **Supplementary Table 6. **Buffers used for flow-cytometry and fluorescence-activated cell sorting. **Supplementary Figure 1. **Glial fibrillary acidic protein is not an optimal marker of ES-astrocyte activation induced by IL-1 α and TNF- α. **Supplementary Figure 2. **Primary culture validation in subventricular zone-astrocytes. **Supplementary Figure 3. **Contact-independent co-culture system of astrocyte-mediated neurotoxicity. **Supplementary Figure 4. **Immunocytochemical depiction of translationally relevant neuroinflammatory mediators. 

## Data Availability

The datasets used and/or analyzed within the current study are available from the corresponding author on reasonable request. For RNA sequencing data, raw data and de-multiplexed raw counts have been deposited in NCBI’s Gene Expression Omnibus [49] and are accessible through GEO Series accession number GSE213804. (https://www.ncbi.nlm.nih.gov/geo/query/acc.cgi?acc=GSE213804) and GEO Series accession number GSE232232 (https://www.ncbi.nlm.nih.gov/geo/query/acc.cgi?acc=GSE232232).

## Abbreviations

AQP4: Aquaporin-4
BMP-4: Bone-morphogenetic protein 4
cAMP: Cyclic adenosine monophosphate
CNS: Central nervous system
CNTF: Ciliary neurotrophic factor
C1Q: Complement component 1Q
C5: Complement component 5
DAPI: 4’,6’-Diamidino-2-phenylindole dihydrochloride
(D)PBS: (Dulbecco’s) phosphate-buffered saline
EB: Embryoid body
EGF: Epidermal growth factor
eGFP: Enhanced green fluorescent protein
ES-: Embryonic stem cell-derived
ES-cells: Embryonic stem cells
FACS: Fluorescence-activated cell sorting
FBS: Fetal bovine serum
FGFb: Basic fibroblast growth factor
FGF1: Acidic fibroblast growth factor
FSK: Forskolin
GFAP: Glial fibrillary acidic protein
GLAST: Glutamate Aspartate Transporter
GLT-1: Glutamate transporter 1
GO: Gene Ontology
IL-: Interleukin-
JNK: C-Jun N-terminal kinase
Hb9: Homeobox gene 9
(h)iPSC: (Human) induced Pluripotent Stem Cells
Nefl: Neurofilament light
NES: Normalized enrichment score
P#: Passage#
P–c-Jun: Phosphorylated c-Jun
PFA: Paraformaldehyde
SVZ: Subventricular zone
TAI: Traumatic Axonal Injury
TBI: Traumatic Brain Injury
TGF: Transforming Growth Factor
TNF: Tumor Necrosis Factor

## References

[1] Freeman MR. Specification and morphogenesis of astrocytes. Science (1979). 2010/11/06. Department of Neurobiology, Howard Hughes Medical Institute, University of Massachusetts Medical School, Worcester, MA 01605, USA. marc.freeman@umassmed.edu; 2010;330:774–8. 10.1126/science.1190928 PMC5201129 21051628

[2] Moulson AJ, Squair JW, Franklin RJM, Tetzlaff W, Assinck P. Diversity of Reactive Astrogliosis in CNS Pathology: Heterogeneity or Plasticity? Front Cell Neurosci. 2021;15. 10.3389/fncel.2021.703810 PMC8349991 34381334

[3] Anderson MA, Ao Y, Sofroniew M V. Heterogeneity of reactive astrocytes. Neurosci Lett. 2013/12/24. Department of Neurobiology, David Geffen School of Medicine, University of California Los Angeles, Los Angeles, CA 90095–1763, United States. Department of Neurobiology, David Geffen School of Medicine, University of California Los Angeles, Los Angeles, C; 2014;565:23–9.

[4] Sofroniew MV (2020). Astrocyte Reactivity: Subtypes, States, and Functions in CNS Innate Immunity. Trends Immunol The Author.

[5] Escartin C, Galea E, Lakatos A, O’Callaghan JP, Petzold GC, Serrano-Pozo A (2021). Reactive astrocyte nomenclature, definitions, and future directions. Nat Neurosci..

[6] Zamanian JL, Xu L, Foo LC, Nouri N, Zhou L, Giffard RG, et al. Genomic analysis of reactive astrogliosis. J Neurosci. 2012/05/04. Department of Neurobiology, Stanford University School of Medicine, Stanford, California 94305–5125, USA. jlz@stanford.edu; 2012;32:6391–410. 10.1523/JNEUROSCI.6221-11.2012 PMC3480225 22553043

[7] Sofroniew MV (2015). Astrocyte barriers to neurotoxic inflammation. Nat Rev Neurosci Nature Publishing Group.

[8] Anderson MA, Ao Y, Sofroniew MV (2014). Heterogeneity of reactive astrocytes. Neurosci Lett Elsevier Ireland Ltd.

[9] Hasel P, Rose IVL, Sadick JS, Kim RD, Liddelow SA (2021). Neuroinflammatory astrocyte subtypes in the mouse brain. Nat Neurosci..

[10] Abu Hamdeh S, Marklund N, Lannsjö M, Howells T, Raininko R, Wikström J (2017). Extended Anatomical Grading in Diffuse Axonal Injury Using MRI: Hemorrhagic Lesions in the Substantia Nigra and Mesencephalic Tegmentum Indicate Poor Long-Term Outcome. J Neurotrauma.

[11] Adams JH, Doyle D, Ford I, Gennarelli TA, Graham DI, Mclellan DR (1989). Diffuse axonal injury in head injury: Definition, diagnosis and grading. Histopathology.

[12] Stahel PF, Kossmann T, Morganti-Kossmann MC, Hans VHJ, Barnum SR (1997). Experimental diffuse axonal injury induces enhanced neuronal C5a receptor mRNA expression in rats. Mole Brain Res Elsevier.

[13] Hans VHJ, Kossmann T, Lenzlinger PM, Probstmeier R, Imhof HG, Trentz O (1999). Experimental axonal injury triggers interleukin-6 mRNA, protein synthesis and release into cerebrospinal fluid. J Cereb Blood Flow Metab.

[14] Medana IM, Esiri MM (2003). Axonal damage: A key predictor of outcome in human CNS diseases. Brain.

[15] Ekmark-Lewén S, Flygt J, Kiwanuka O, Meyerson BJ, Lewén A, Hillered L (2013). Traumatic axonal injury in the mouse is accompanied by a dynamic inflammatory response, astroglial reactivity and complex behavioral changes. J Neuroinflammation.

[16] Csuka E, Hans VHJ, Ammann E, Trentz O, Kossmann T, Morganti-Kossmann MC (2000). Cell activation and inflammatory response following traumatic axonal injury in the rat. NeuroReport.

[17] Liddelow SA, Guttenplan KA, Clarke LE, Bennett FC, Bohlen CJ, Schirmer L (2017). Neurotoxic reactive astrocytes are induced by activated microglia. Nature..

[18] Clarke BE, Taha DM, Ziff OJ, Alam A, Thelin EP, Garcia NM (2020). Human stem cell-derived astrocytes exhibit region-specific heterogeneity in their secretory profiles. Brain.

[19] Barbar L, Jain T, Zimmer M, Kruglikov I, Sadick JS, Wang M (2020). CD49f Is a Novel Marker of Functional and Reactive Human iPSC-Derived Astrocytes. Neuron.

[20] Nagai M, Re DB, Nagata T, Chalazonitis A, Jessell TM, Wichterle H (2007). Astrocytes expressing ALS-linked mutated SOD1 release factors selectively toxic to motor neurons. Nat Neurosci.

[21] Statens jordbruksverks författningssamling [Internet]. 2019 [cited 2020 Mar 13]. Available from: http://www.jordbruksverket.se/download/18.7c1e1fce169bee5214fad877/1553851490782/2019-009.pdf

[22] Wichterle H, Lieberam I, Porter JA, Jessell TM. Directed differentiation of embryonic stem cells into motor neurons. Cell. 2002/08/15. Howard Hughes Medical Institute, Department of Biochemistry and Molecular Biophysics, Columbia University, New York, NY 10032, USA.; 2002;110:385–97. 10.1016/s0092-8674(02)00835-8 12176325

[23] Kleiderman S, Sá JV, Teixeira AP, Brito C, Gutbier S, Evje LG (2016). Functional and phenotypic differences of pure populations of stem cell-derived astrocytes and neuronal precursor cells. Glia.

[24] Roybon L, Lamas NJ, Garcia-Diaz A, Yang EJ, Sattler R, Jackson-Lewis V (2013). Human stem cell-derived spinal cord astrocytes with defined mature or reactive phenotypes. Cell Rep The Authors.

[25] Kim BJ, Kim SS, Kim YI, Paek SH, Lee YD, Suh-Kim H (2004). Forskolin promotes astroglial differentiation of human central neurocytoma cells. Exp Mol Med.

[26] McManus MF, Chen LC, Vallejo I, Vallejo M. Astroglial differentiation of cortical precursor cells triggered by activation of the cAMP-dependent signaling pathway. J Neurosci. 1999/10/12. Reproductive Endocrine Unit, Massachusetts General Hospital, Harvard Medical School, Boston, Massachusetts 02114, USA.; 1999;19:9004–15. 10.1523/JNEUROSCI.19-20-09004.1999 PMC6782746 10516318

[27] Rajan P, McKay RDG (1998). Multiple routes to astrocytic differentiation in the CNS. J Neurosci.

[28] Bonni A, Sun Y, Nadal-Vicens M, Bhatt A, Frank DA, Rozovsky I, et al. Regulation of gliogenesis in the central nervous system by the JAK-STAT signaling pathway. Science (1979). 1997/10/23. Division of Neuroscience, Children’s Hospital, and Department of Neurobiology, Harvard Medical School, Boston, MA 02115, USA.; 1997;278:477–83. 10.1126/science.278.5337.477 9334309

[29] Kitchen P, Salman MM, Halsey AM, Clarke-Bland C, MacDonald JA, Ishida H (2020). Targeting Aquaporin-4 Subcellular Localization to Treat Central Nervous System Edema. Cell.

[30] Johansson CB, Momma S, Clarke DL, Risling M, Lendahl U, Frisén J (1999). Identification of a neural stem cell in the adult mammalian central nervous system. Cell Cell Press.

[31] Covacu R, Danilov AI, Rasmussen BS, Hallén K, Moe MC, Lobell A (2006). Nitric Oxide Exposure Diverts Neural Stem Cell Fate from Neurogenesis Towards Astrogliogenesis. Stem Cells Wiley.

[32] Burda JE, Bernstein AM, Sofroniew M V. Astrocyte roles in traumatic brain injury. Exp Neurol. Elsevier Inc.; 2016;275:305–15. 10.1016/j.expneurol.2015.03.020 PMC4586307 25828533

[33] Lindblad C, Pin E, Just D, Al Nimer F, Nilsson P, Bellander B-M, et al. Fluid Proteomics of CSF and Serum Reveal Important Neuroinammatory Proteins in Blood-Brain Barrier Disruption and Outcome Prediction Following Severe Traumatic Brain Injury: A Prospective, Observational Study. Crit Care [Internet]. BioMed Central; 2021;1–28. Available from: 10.21203/rs.3.rs-96625/v1 10.1186/s13054-021-03503-x PMC7955664 33712077

[34] Okusawa BYS, W KIMBYJOS, Meer MVANDER, Endres S, Lonnemann G, Hefter K, et al. C5a STIMULATES SECRETION OF TUMOR NECROSIS Comparison with Secretion of Interleukin 10 and Interleukin la. 1988;168. 10.1084/jem.168.1.443 PMC2188974 3260938

[35] Webster RO, Hong SR, Johnston RB, Henson PM. Biological Effects of the Human Complement Fragments C5a and C5ades Arg on Neutrophil Function. 1980;219:201–19. 10.1016/0162-3109(80)90050-8 6254906

[36] Thelin EP, Hall CE, Gupta K, Carpenter KLH, Chandran S, Hutchinson PJ (2018). Elucidating Pro-Inflammatory Cytokine Responses after Traumatic Brain Injury in a Human Stem Cell Model. J Neurotrauma.

[37] Thelin EP, Hall CE, Tyzack GE, Frostell A, Giorgi-Coll S, Alam A (2020). Delineating Astrocytic Cytokine Responses in a Human Stem Cell Model of Neural Trauma. J Neurotrauma.

[38] Skiljan I. IrfanView - Official Homepage - One of the Most Popular Viewers Worldwide [Internet]. [cited 2021 Nov 22]. Available from: https://www.irfanview.com/

[39] Berglund R, Guerreiro-Cacais AO, Adzemovic MZ, Zeitelhofer M, Lund H, Ewing E, et al. Microglial autophagy-associated phagocytosis is essential for recovery from neuroinflammation. Sci Immunol. 2020;5. 10.1126/sciimmunol.abb5077 33067381

[40] Hedlund E, Pruszak J, Lardaro T, Ludwig W, Viñuela A, Kim K-S (2008). Embryonic Stem Cell-Derived Pitx3-Enhanced Green Fluorescent Protein Midbrain Dopamine Neurons Survive Enrichment by Fluorescence-Activated Cell Sorting and Function in an Animal Model of Parkinson’s Disease. Stem Cells.

[41] Allodi I, Nijssen J, Benitez JA, Schweingruber C, Fuchs A, Bonvicini G (2019). Modeling Motor Neuron Resilience in ALS Using Stem Cells. Stem Cell Reports ElsevierCompany.

[42] Zachariadis V, Cheng H, Andrews N, Enge M. A Highly Scalable Method for Joint Whole-Genome Sequencing and Gene-Expression Profiling of Single Cells. Mol Cell. 2020;80:541–553.e5. Elsevier Inc. 10.1016/j.molcel.2020.09.025 33068522

[43] Zachariadis V, Cheng H, Andrews N, Enge M. Direct nuclear tagmentation and RNA-sequencing ( DNTR- seq ). protocols.io. 2020;

[44] Zhu YY, Machleder EM, Chenchik A, Li R, Siebert PD (2001). Reverse transcriptase template switching: A SMART™ approach for full-length cDNA library construction. Biotechniques.

[45] Ramsköld D, Luo S, Wang YC, Li R, Deng Q, Faridani OR (2012). Full-length mRNA-Seq from single-cell levels of RNA and individual circulating tumor cells. Nat Biotechnol.

[46] Picelli S, Faridani OR, Björklund ÅK, Winberg G, Sagasser S, Sandberg R (2014). Full-length RNA-seq from single cells using Smart-seq2. Nat Protoc.

[47] Adey A, Morrison HG, Asan, Xun X, Kitzman JO, Turner EH, et al. Rapid, low-input, low-bias construction of shotgun fragment libraries by high-density in vitro transposition. Genome Biol. 2010;11. 10.1186/gb-2010-11-12-r119 PMC3046479 21143862

[48] Dobin A, Davis CA, Schlesinger F, Drenkow J, Zaleski C, Jha S (2013). STAR: Ultrafast universal RNA-seq aligner. Bioinformatics.

[49] Edgar R, Domrachev M, Lash AE (2002). Gene Expression Omnibus: NCBI gene expression and hybridization array data repository. Nucleic Acids Res.

[50] Team RC. R: A language and environment for statistical computing. Vienna, Austria: R Foundation for Statistical Computing; 2018.

[51] Neuwirth E. RColorBrewer: ColorBrewer Palettes. 2014.

[52] Wilke CO. cowplot: Streamlined Plot Theme and Plot Annotations for “ggplot2.” Comprehensive R Archive Network (CRAN); 2019.

[53] Auguie B. gridExtra: Miscellaneous Functions for “Grid” Graphics. Comprehensive R Archive Network (CRAN); 2017.

[54] Wickham H, Averick M, Bryan J, Chang W, McGowan L, François R (2019). Welcome to the Tidyverse. J Open Source Softw.

[55] Fox J, Weisberg S (2019). An R Companion to Applied Regression.

[56] Ogle DH, Doll JC, Wheeler P, Dinno A. FSA: Fisheries Stock Analysis. R package; 2021.

[57] Dag O, Dolgun A, Meric Konar N. onewaytests: An R Package for One-Way Tests in Independent Groups Designs.

[58] Durinck S, Moreau Y, Kasprzyk A, Davis S, De Moor B, Brazma A (2005). BioMart and Bioconductor: A powerful link between biological databases and microarray data analysis. Bioinformatics.

[59] Durinck S, Spellman PT, Birney E, Huber W (2009). Mapping identifiers for the integration of genomic datasets with the R/ Bioconductor package biomaRt. Nat Protoc.

[60] Love MI, Huber W, Anders S (2014). Moderated estimation of fold change and dispersion for RNA-seq data with DESeq2. Genome Biol.

[61] Love M, Anders S, Huber W (2017). Analyzing RNA-seq data with DESeq2. Bioconductor.

[62] John CR, Watson D, Russ D, Goldmann K, Ehrenstein M, Pitzalis C (2020). M3C: Monte Carlo reference-based consensus clustering. Sci Rep.

[63] Witten D. PoiClaClu: Classification and Clustering of Sequencing Data Based on a Poisson Model. CRAN, R package; 2019.

[64] Zhu A, Ibrahim JG, Love MI (2019). Heavy-Tailed prior distributions for sequence count data: Removing the noise and preserving large differences. Bioinformatics.

[65] Subramanian A, Tamayo P, Mootha VK, Mukherjee S, Ebert BL, Gillette MA (2005). Gene set enrichment analysis: A knowledge-based approach for interpreting genome-wide expression profiles. Proc Natl Acad Sci U S A.

[66] Korotkevich G, Sukhov V, Budin N, Shpak B, Artyomov M, Sergushichev A. Fast gene set enrichment analysis. 2019;

[67] Liberzon A, Birger C, Thorvaldsdóttir H, Ghandi M, Mesirov JP, Tamayo P (2015). The Molecular Signatures Database Hallmark Gene Set Collection. Cell Syst.

[68] Jassal B, Matthews L, Viteri G, Gong C, Lorente P, Fabregat A (2020). The reactome pathway knowledgebase. Nucleic Acids Res Oxford University Press.

[69] Consortium TGO. Gene Ontology : tool for the unification of biology. Nat Genet. 2000;25:25–9. 10.1038/75556 PMC3037419 10802651

[70] Kanehisa M (2000). KEGG: Kyoto Encyclopedia of Genes and Genomes. Nucleic Acids Res.

[71] Liberzon A, Subramanian A, Pinchback R, Thorvaldsdóttir H, Tamayo P, Mesirov JP (2011). Molecular signatures database (MSigDB) 3.0. Bioinformatics..

[72] Dolgalev I. msigdbr: MSigDB Gene Sets for Multiple Organisms in a Tidy Data Format. CRAN, R package; 2021.

[73] Carlson M. GO.db: A set of annotation maps describing the entire Gene Ontology. Bioconductor, R package; 2021.

[74] Rowitch DH, Kriegstein AR (2010). Developmental genetics of vertebrate glial-cell specification. Nature.

[75] Zuchero JB, Barres BA (2015). Glia in mammalian development and disease. Development (Cambridge).

[76] Allodi I, Hedlund E (2014). Directed midbrain and spinal cord neurogenesis from pluripotent stem cells to model development and disease in a dish. Front Neurosci.

[77] Ye ZC, Sontheimer H (1998). Astrocytes protect neurons from neurotoxic injury by serum glutamate. Glia.

[78] Eng LF, Ghirnikar RS, Lee YL (2000). Glial Fibrillary Acidic Protein: GFAP-Thirty-One Years (1969–2000)*. Neurochem Res.

[79] Roybon L, Lamas NJ, Garcia-Diaz A, Yang EJ, Sattler R, Jackson-Lewis V, et al. Human stem cell-derived spinal cord astrocytes with defined mature or reactive phenotypes. Cell Rep. 2013/09/03. Project A.L.S./Jenifer Estess Laboratory for Stem Cell Research, Columbia University Medical Center, P&S 16–440, 630 West 168(th) Street, New York, NY 10032, USA Columbia Stem Cell Initiative (CSCI), Departments of Pathology and Cell Biology and Neurology; 2013;4:1035–48. 10.1016/j.celrep.2013.06.021 PMC4229657 23994478

[80] Molofsky A V, Krenick R, Ullian E, Tsai H -h., Deneen B, Richardson WD, et al. Astrocytes and disease: a neurodevelopmental perspective. Genes Dev. 2012;26:891–907. 10.1101/gad.188326.112 PMC3347787 22549954

[81] Regan MR, Huang YH, Yu SK, Dykes-Hoberg MI, Jin L, Watkins AM (2007). Variations in promoter activity reveal a differential expression and physiology of glutamate transporters by glia in the developing and mature CNS. J Neurosci.

[82] Abbott NJ, Rönnbäck L, Hansson E (2006). Astrocyte-endothelial interactions at the blood-brain barrier. Nat Rev Neurosci.

[83] Philippidou P, Dasen JS (2013). Hox Genes: Choreographers in Neural Development. Architects of Circuit Organization. Neuron..

[84] Krencik R, Weick JP, Liu Y, Zhang ZJ, Zhang SC (2011). Specification of transplantable astroglial subtypes from human pluripotent stem cells. Nat Biotechnol Nature Publishing Group.

[85] Lozzi B, Huang TW, Sardar D, Huang AYS, Deneen B. Regionally Distinct Astrocytes Display Unique Transcription Factor Profiles in the Adult Brain. Front Neurosci. Frontiers Media S.A.; 2020;14. 10.3389/fnins.2020.00061 PMC7046629 32153350

[86] Hochstim C, Deneen B, Lukaszewicz A, Zhou Q, Anderson DJ (2008). Identification of Positionally Distinct Astrocyte Subtypes whose Identities Are Specified by a Homeodomain Code. Cell.

[87] Gaetani L, Blennow K, Calabresi P, Di Filippo M, Parnetti L, Zetterberg H (2019). Neurofilament light chain as a biomarker in neurological disorders.

[88] Khalil M, Teunissen CE, Otto M, Piehl F, Sormani MP, Gattringer T, et al. Neurofilaments as biomarkers in neurological disorders. Nat Rev Neurol. Nature Publishing Group; 2018. p. 577–89. 10.1038/s41582-018-0058-z 30171200

[89] Thams S, Lowry ER, Larraufie MH, Spiller KJ, Li H, Williams DJ (2019). A Stem Cell-Based Screening Platform Identifies Compounds that Desensitize Motor Neurons to Endoplasmic Reticulum Stress. Molecular Therapy Cell Press.

[90] Pfaff SL, Mendelsohn M. Requirement for LIM Homeobox Gene Isl1 in Motor Neuron Generation Reveals a Motor Neuron-Dependent Step in Interneuron Differentiation. Cell. 1996. 10.1016/s0092-8674(00)80985-x 8565076

[91] Cahoy JD, Emery B, Kaushal A, Foo LC, Zamanian JL, Christopherson KS (2008). A Transcriptome Database for Astrocytes, Neurons, and Oligodendrocytes: A New Resource for Understanding Brain Development and Function. J Neurosci.

[92] Pekny M, Wilhelmsson U, Pekna M (2014). The dual role of astrocyte activation and reactive gliosis. Neurosci Lett Elsevier Ireland Ltd.

[93] Weber A, Wasiliew P, Kracht M. Interleukin-1 (IL-1) Pathway. Sci Signal. 2010;3:cm1–cm1. 10.1126/scisignal.3105cm1 20086235

[94] Zhang P, Miller BS, Rosenzweig SA, Bhat NR (1996). Activation of c-Jun N-terminal kinase/stress-activated protein kinase in primary glial cultures. J Neurosci Res.

[95] Zeke A, Misheva M, Reményi A, Bogoyevitch MA (2016). JNK Signaling: Regulation and Functions Based on Complex Protein-Protein Partnerships. Microbiol Mol Biol Rev.

[96] Schreck I, Al-Rawi M, Mingot JM, Scholl C, Diefenbacher ME, O’Donnell P (2011). C-Jun localizes to the nucleus independent of its phosphorylation by and interaction with JNK and vice versa promotes nuclear accumulation of JNK. Biochem Biophys Res Commun..

[97] Jochum W, Passegué E, Wagner EF (2001). AP-1 in mouse development and tumorigenesis. Oncogene.

[98] Raivich G. c-Jun Expression, activation and function in neural cell death, inflammation and repair. J Neurochem. 2008. p. 898–906. 10.1111/j.1471-4159.2008.05684.x 18793328

[99] Gao K, Wang CR, Jiang F, Wong AY, Su N, Jiang JH, et al. Traumatic scratch injury in astrocytes triggers calcium influx to activate the JNK/c-Jun/AP-1 pathway and switch on GFAP expression. Glia. 2013/10/15. Neuroscience Research Institute, Key Laboratory for Neuroscience (Ministry of Education), Key Laboratory for Neuroscience (National Health and Family Planning Commission), Department of Neurobiology, School of Basic Medical Sciences, Health Science Center; 2013;61:2063–77.

[100] Iram T, Ramirez-Ortiz Z, Byrne MH, Coleman UA, Kingery ND, Means TK (2016). Megf10 Is a receptor for C1Q that mediates clearance of apoptotic cells by astrocytes. J Neurosci.

[101] MacDonald JM, Doherty J, Hackett R, Freeman MR (2013). The c-Jun kinase signaling cascade promotes glial engulfment activity through activation of draper and phagocytic function. Cell Death Differ Nature Publishing Group.

[102] Doetsch F, Caillé I, Lim DA, García-Verdugo JM, Alvarez-Buylla A (1999). Subventricular Zone Astrocytes Are Neural Stem Cells in the Adult Mammalian Brain. Cell.

[103] Covacu R, Estrada CP, Arvidsson L, Svensson M, Brundin L (2014). Change of fate commitment in adult neural progenitor cells subjected to chronic inflammation. J Neurosci.

[104] Molofsky A V, Deneen B. Astrocyte development: A Guide for the Perplexed. Glia. 2015/05/13. Department of Psychiatry, University of California-San Francisco, San Francisco, California. Center for Cell and Gene Therapy, Baylor College of Medicine, Houston, Texas.; 2015;63:1320–9. 10.1002/glia.22836 25963996

[105] Middeldorp J, Hol EM (2011). GFAP in health and disease. Prog Neurobiol Elsevier Ltd.

[106] Woodcock T, Morganti-Kossmann MC. The role of markers of inflammation in traumatic brain injury. Front Neurol. 2013;4 MAR:1–18. 10.3389/fneur.2013.00018 PMC3586682 23459929

[107] Helmy A, De Simoni MG, Guilfoyle MR, Carpenter KLH, Hutchinson PJ. Cytokines and innate inflammation in the pathogenesis of human traumatic brain injury. Prog Neurobiol. 2011;95:352–72. [Internet]. Elsevier Ltd;10.1016/j.pneurobio.2011.09.003 10.1016/j.pneurobio.2011.09.003 21939729

[108] Abu Hamdeh S, Shevchenko G, Mi J, Musunuri S, Bergquist J, Marklund N (2018). Proteomic differences between focal and diffuse traumatic brain injury in human brain tissue. Sci Rep.

[109] Sloan SA, Darmanis S, Huber N, Khan TA, Birey F, Caneda C (2017). Human Astrocyte Maturation Captured in 3D Cerebral Cortical Spheroids Derived from Pluripotent Stem Cells. Neuron.

[110] Canals I, Ginisty A, Quist E, Timmerman R, Fritze J, Miskinyte G (2018). Rapid and efficient induction of functional astrocytes from human pluripotent stem cells. Nat Methods.

[111] Westergard T, Rothstein JD (2020). Astrocyte Diversity: Current Insights and Future Directions. Neurochem Res..

[112] Zhang Y, Barres BA (2010). Astrocyte heterogeneity: an underappreciated topic in neurobiology. Curr Opin Neurobiol.

[113] Tsai HH, Li H, Fuentealba LC, Molofsky AV, Taveira-Marques R, Zhuang H (1979). Regional astrocyte allocation regulates CNS synaptogenesis and repair. Science.

[114] Morel L, Chiang MSR, Higashimori H, Shoneye T, Iyer LK, Yelick J (2017). Molecular and functional properties of regional astrocytes in the adult brain. J Neurosci.

[115] Werner C, Engelhard K (2007). Pathophysiology of traumatic brain injury. Br J Anaesth..

[116] Kumar A, Loane DJ. Neuroinflammation after traumatic brain injury: Opportunities for therapeutic intervention. Brain Behav Immun [Internet]. Elsevier Inc.; 2012;26:1191–201. Available from: 10.1016/j.bbi.2012.06.008 10.1016/j.bbi.2012.06.008 22728326

[117] Foo LC, Allen NJ, Bushong EA, Ventura PB, Chung WS, Zhou L (2011). Development of a method for the purification and culture of rodent astrocytes. Neuron.

[118] Zhang Y, Sloan SA, Clarke LE, Caneda C, Plaza CA, Blumenthal PD (2016). Purification and Characterization of Progenitor and Mature Human Astrocytes Reveals Transcriptional and Functional Differences with Mouse. Neuron Cell Press.

[119] Perriot S, Mathias A, Perriard G, Canales M, Jonkmans N, Merienne N (2018). Human Induced Pluripotent Stem Cell-Derived Astrocytes Are Differentially Activated by Multiple Sclerosis-Associated Cytokines. Stem Cell Reports Cell Press.

[120] Khan SA, Joyce J, Tsuda T. Quantification of active and total transforming growth factor-β levels in serum and solid organ tissues by bioassay. BMC Res Notes. 2012;5. 10.1186/1756-0500-5-636 PMC3556312 23151377

[121] Oida T, Weiner HL (2010). Depletion of TGF-β from fetal bovine serum. J Immunol Methods.

[122] Guttenplan KA, Weigel MK, Prakash P, Wijewardhane PR, Hasel P, Rufen-Blanchette U, et al. Neurotoxic reactive astrocytes induce cell death via saturated lipids. Nature. 2021; 10.1038/s41586-021-03960-y PMC12054010 34616039

[123] Li J, Pan L, Pembroke WG, Rexach JE, Godoy MI, Condro MC, et al. Conservation and divergence of vulnerability and responses to stressors between human and mouse astrocytes. Nat Commun [Internet]. Springer US; 2021;12:1–20. Available from: 10.1038/s41467-021-24232-3 10.1038/s41467-021-24232-3 PMC8233314 34172753

[124] Bush TG, Puvanachandra N, Horner CH, Polito A, Ostenfeld T, Svendsen CN (1999). Leukocyte infiltration, neuronal degeneration, and neurite outgrowth after ablation of scar-forming, reactive astrocytes in adult transgenic mice. Neuron.

[125] Anderson MA, Burda JE, Ren Y, Ao Y, O’Shea TM, Kawaguchi R, et al. Astrocyte scar formation aids central nervous system axon regeneration. Nature. 2016/03/31. Department of Neurobiology, David Geffen School of Medicine, University of California, Los Angeles, California 90095–1763, USA. Departments of Psychiatry and Neurology, David Geffen School of Medicine, University of California, Los Angeles, California 900; 2016;532:195–200. 10.1038/nature17623 PMC5243141 27027288

[126] Li Z, Xiao J, Xu X, Li W, Zhong R, Qi L, et al. M-CSF, IL-6, and TGF-β promote generation of a new subset of tissue repair macrophage for traumatic brain injury recovery. Sci Adv. 2021;7. 10.1126/sciadv.abb6260 PMC7954455 33712456

[127] Yang SH, Gangidine M, Pritts TA, Goodman MD, Lentsch AB (2013). Interleukin 6 mediates neuroinflammation and motor coordination deficits after mild traumatic brain injury and brief hypoxia in mice. Shock.

