# Supplementary material for: Stem cell-derived brainstem mouse astrocytes obtain a neurotoxic phenotype in vitro upon neuroinflammation
Source: J Inflamm (Lond). 2023 Jun 27;20:22. doi: 10.1186/s12950-023-00349-8 (PMC10303821; doi:10.1186/s12950-023-00349-8)
Supplement: Supplementary file 1 — Additional file 1: Supplementary Methods; Supplementary References. Supplementary Table 1. Media constituents for motor neuron differentiation. Supplementary Table 2. Media constituents for astrocyte differentiation. Supplementary Table 3. Media constituents for primary SVZ cell culture. Supplementary Table 4. Neuroinflammatory factors used. Supplementary Table 5. Materials used for immunocytochemistry. Supplementary Table 6. Buffers used for flow-cytometry and fluorescence-activated cell sorting. Supplementary Figure 1. Glial fibrillary acidic protein is not an optimal marker of ES-astrocyte activation induced by IL-1 α and TNF- α. Supplementary Figure 2. Primary culture validation in subventricular zone-astrocytes. Supplementary Figure 3. Contact-independent co-culture system of astrocyte-mediated neurotoxicity. Supplementary Figure 4. Immunocytochemical depiction of translationally relevant neuroinflammatory mediators. [file 12950_2023_349_MOESM1_ESM.zip › supplementaryText_230622_CL_ESM.docx]

**Supplementary Material**

**Stem cell-derived brainstem mouse astrocytes obtain a neurotoxic phenotype *in vitro* upon neuroinflammation**

Caroline Lindblad^1*^, Susanne Neumann^1^, Sólrún Kolbeinsdóttir^2^, Vasilios Zachariadis^2^, Eric P Thelin^1,3^, Martin Enge^2^, Sebastian Thams^1,3^, Lou Brundin^1,3^, Mikael Svensson^1,4^

1: Department of Clinical Neuroscience, Karolinska Institutet, Stockholm, Sweden.

2: Department of Oncology-Pathology, Karolinska Institutet, Stockholm, Sweden.

3: Department of Neurology Karolinska University Hospital, Stockholm, Sweden.

4: Department of Neurosurgery Karolinska University Hospital, Stockholm, Sweden.

***Correspondence:**

Caroline Lindblad

Department of Clinical Neuroscience

J5:20 Svensson Group, Karolinska Universitetssjukhuset Solna

SE-171 77 Stockholm, SWEDEN

[caroline.lindblad@ki.se](mailto:caroline.lindblad@ki.se)

+46708-236282

Supplementary Methods

**Directed differentiation of embryonic stem cells into brainstem motor neurons**

ES cells were seeded in T25 flasks (VWR) coated with 0.1 % gelatin (Merck Millipore) in ES medium **(Supplementary Table 1)**. Following two days of expansion, differentiation was induced by dissociating ES cells using 0.05 % trypsin (Gibco). Cells were collected, centrifuged at 400 rcf, and subsequently replated as single-cells in differentiation medium **(Supplementary Table 1)** on semiadhesive plates (VWR). One day following this procedure, neural spheres (embryoid bodies [EBs]) emerged, were collected, and plated in fresh differentiation medium. Two days after initiation of differentiation, EBs were reseeded in differentiation medium supplemented with 1μM retinoic acid and 0.5 μM smoothened agonist in non-treated plates (Corning). On day five, medium was replaced with fresh differentiation medium, supplemented with only 10 ng/mL glial cell-line derived neurotrophic factor.

On day six, EBs were dissociated into single cell populations of neurons and glial progenitor cells. EBs were collected, and washed using 0.8% 45%-glucose (Sigma-Aldrich) diluted in 1X Dulbecco’s phosphate buffered saline (Thermo-Fisher Scientific) (DPBS-glucose). EBs were dissociated using 0.05% trypsin (Gibco) and trituration. Trypsinization was inhibited using sterile-filtered fetal bovine serum (FBS, **Supplementary** **Table 1**). Dissociated cells were centrifuged (400 rcf, 5 mins) and filtered through a 40 μm cell strainer (fisherbrand). Cell number was assessed and cells were resuspended in either motor neuron medium **(Supplementary Table 1)** or as described below for other down-stream assays.

**RNA extraction from subventricular zone stem cells**

Twenty-four hours after plating, subventricular zone stem cells (see **Primary culture validation using subventricular zone stem cells**) were supplemented with 100µl PBS/1ml culture medium and incubated for one and eight hours. The cells were subsequently detached from the PDL-coated wells using a Cell Scraper and RLT- lysis buffer supplemented with 1% β-mercaptoethanol. Meanwhile, the cells were mechanically dissociated with pipetting the suspension harshly up and down. Total RNA was isolated using the RNeasy mini kit (Qiagen 74104) according to the manufacturer’s recommendations, contaminating genomic DNA was eliminated by on-column digestion using DNase I (Qiagen 79254). The RNA was stored at -70^◦^C until analysis.

**Bulk RNA sequencing of undifferentiated subventricular zone stem cells**

RNA sequencing was performed by the National Genomics Infrastructure (NGI) Sweden at Scilife, Stockholm. Strand-specific Illumina TruSeq RNA libraries using poly-A selection were prepared for n = 5 total RNA samples from isolated subventricular zones. Quality analysis was performed using Bioanalyzer (conc. >250 ng, RIN>9). Libraries were sequenced using Multiplex in 3 lanes of Illumina HiSeq 2500 (HiSeq Control Software 2.2.58/RTA 1.18.64) high Output V4, PE 2x125bp. Reads were mapped against the reference genome Mouse (Mus Musculus, GRCm38). The raw sequence data consisted of sequence reads in FastQ format, using the Phred33/Sanger/Illumina 1.8+ encoding (Bcl to FastQ conversion was done using bcl2fastq_v2.19.1.403 from the CASAVA software suite). Analysis was performed using the nf-core/rnaseq best-practice analysis pipeline. Raw data and processed files for subsequent analysis was delivered to on Uppmax/GRUS (DELIVERY_P9913_2019-01-09; <https://supr.snic.se/project/10596/>). Using STAR feature counts and Ensembl annotation release a summary of read counts over genes was obtained. For RNA sequencing data, raw data and de-multiplexed raw counts have been deposited in NCBI’s Gene Expression Omnibus [1]and are accessible through GEO Series accession number GSE232232 (<https://www.ncbi.nlm.nih.gov/geo/query/acc.cgi?acc=GSE232232>).

Supplementary Tables

| **Supplementary Table 1: Media constituents for motor neuron differentiation** | | | | |
| --- | --- | --- | --- | --- |
| **ES medium** | **Constituent** | **Concentration (% of total solution)** | **Manufacturer** | **Article number** |
|  | EmbryoMax | 80% | EMD Millipore | SLM-220-B |
|  | Fetal bovine serum | 15% | Thermo Fisher Scientific | 10082-147 |
|  | L-glutamine 200 mM | 1% | Thermo Fisher Scientific | 25030081 |
|  | EmbryoMax® Nucleosides | 1% | Millipore Sigma | ES-008-D |
|  | EmbryoMax® Non-essential aminoacids | 1% | Millipore Sigma | TMS-001-C |
|  | β -mercaptoethanol | 0.07 % of a 1% solution | Acros | 125472500 |
|  | Penicillin-streptomycin | 1% | Thermo Fisher Scientific | 15140122 |
|  | LIF | 0.01% | EMD Millipore | ESG1107 |
| **Differentiation medium** | **Constituent** | **Concentration (of total solution)** | **Manufacturer** | **Article number** |
|  | Advanced DMEM/F12 | 44% | Thermo Fisher Scientific | 12634-028 |
|  | Neurobasal | 44% | Thermo Fisher Scientific | 21103-049 |
|  | Knockout serum replacement | 10% | Gibco | 10828-028 |
|  | L-glutamine | 1% | Thermo Fisher Scientific | 25030081 |
|  | β -mercaptoethanol | 0.07% of a 1% solution | Acros | 125472500 |
|  | Penicillin-streptomycin | 1% | Thermo Fisher Scientific | 15140122 |
| **MN medium** | **Constituent** | **Concentration (of total solution)** | **Manufacturer** | **Article number** |
|  | Neurobasal | 95.5% | Thermo Fisher Scientific | 21103-049 |
|  | L-glutamine | 0.25 % | Thermo Fisher Scientific | 25030081 |
|  | B-27 | 2% | Thermo Fisher Scientific | 17504-044 |
|  | β -mercaptoethanol | 0.07% of a 1% solution | Acros | 125472500 |
|  | Fetal bovine serum | 2% | Thermo Fisher Scientific | 10082-147 |
|  | Penicillin-streptomycin | 1% | Thermo Fisher Scientific | 15140122 |
| **Supplements** | **Constituent** | **Concentration (in final solution)** | **Manufacturer** | **Article number** |
|  | Retinoic acid (RA) | 1 μM | Sigma-Aldrich | R2625 |
|  | Sonic hedgehog agonist (SAG) | 0.5 μM | EMD Millipore | 566660-1mg |
|  | Glial cell-derived neurotrophic factor (GDNF); recombinant human | 10 ng/mL | R&D | 212GD-010 |
| **Additional buffers or material** | **Constituent** | **Concentration (in final solution)** | **Manufacturer** | **Article number** |
|  | Trypsin | 0.05% | Gibco | 15400-054 |
|  | Gelatin | 0.1% | Merck Millipore | ES-006-B |
|  | Mouse irradiated fibroblasts | NA | Thermo Fisher Scientific | A34180 |
|  | Cell strainer (40 μM) | NA | Fisherbrand | 22363547 |
|  | Easy flask/T25, Nunc^TM^ Δ coated | NA | VWR | 734-2004 |
|  | Cell culture dish, Nunc^TM^ Δ coated, Ø 10 cm | NA | VWR | 734-2704 |
|  | Corning® Cell culture dishes, non-treated | NA | Sigma-Aldrich | CLS430591-100EA |
|  | Cell counter | NA | Bio-Rad | TC20^TM^ |
| Above, media constituents and additional material needed for motor neuron differentiation from embryonic stem cells are provided. Abbreviations: ES, embryonic stem cell; MN, motor neuron. | | | | |

| **Supplementary Table 2: Media constituents for astrocyte differentiation** | | | | |
| --- | --- | --- | --- | --- |
| **Astrocyte-medium** | **Constituent** | **Concentration (of total solution)** | **Manufacturer** | **Article number** |
|  | Advanced DMEM/F12 | 87% | Thermo Fisher Scientific | 12634-028 |
|  | B27 | 2% | Thermo Fisher Scientific | 17504-044 |
|  | Fetal bovine serum | 10% | Thermo Fisher Scientific | 10082-147 |
|  | Penicillin-streptomycin | 1% | Thermo Fisher Scientific | 15140122 |
| **Supplemental factors** | **Constituent** | **Concentration (in final solution)** | **Manufacturer** | **Article number** |
|  | Recombinant rat fibroblast growth factor (FGF) basic | 25 ng/mL | R&D | 3339-FB |
|  | Recombinant human epidermal growth factor (EGF) | 20 ng/mL | R&D | 236-EG-200 |
| **Differentiation factors** | **Constituent** | **Concentration (in final solution)** | **Manufacturer** | **Article number** |
|  | Recombinant human Bone morphogenetic protein 4 (BMP-4) | 20 ng/mL | R&D | 314-BP-010 |
|  | Ciliary neurotrophic factor (CNTF) | 10 ng/mL | Invitrogen | PRC7015 |
|  | Fibroblast growth factor 1 (acidic) | 50 ng/mL | R&D | 231-BC |
|  | Forskolin | 10 μM | Sigma-Aldrich | F6886 |
| **Additional buffers or material** | **Constituent** | **Concentration (in final solution)** | **Manufacturer** | **Article number** |
|  | 96 well glass bottom plates | NA | Greiner Bio-One | 655090 |
|  | Cell culture dish, Nunc^TM^ Δ coated | NA | VWR | 734-2704 |
|  | Poly-L-ornithine hydrobromide | 100 μg/mL | Sigma-Aldrich | P3655 |
|  | Laminin (mouse) | 1 μg/mL | Thermo Fisher Scientific | 23017015 |
| Above, media constituents and additional material needed for astrocyte differentiation from glial progenitors are provided. | | | | |

| **Supplementary Table 3: Media constituents for primary SVZ cell culture** | | | | |
| --- | --- | --- | --- | --- |
| **Dissociation Medium** | **Constituent** | **Concentration** | **Manufacturer** | **Article number** |
|  | Leibovitz's L-15 Medium, GlutaMAX™ Supplement |  | **Gibco™** | 31415086 |
|  | Papain | 1:100 (0,454 mg/ml) | Worthington Biochemical Corporation | LS003126 |
|  | Filter used | Syringe Filters, Filtropur S 0.2 | Sarsted | 103573-246 |
| **Expansion Medium** | **Constituent** | **Concentration** | **Manufacturer** | **Article number** |
|  | DMEM/F-12, GlutaMAX™ Supplement | 2 % | Gibco™ | 31331093 |
|  | Penicillin-Streptomycin | 0.01% | Sigma-Aldrich | P4458 |
|  | B27 Supplement | 2 % | Gibco™ | 17504044 |
|  | Epidermal Growth Factor from murine submaxillary gland | 20 ng/mL | Sigma-Aldrich | E4127 |
|  | Recombinant Human FGF basic/FGF2/bFGF Protein | 5 ng/mL | R&D systems | 233-FB |
| **Additives** | **Constituent** | **Concentration** | **Manufacturer** | **Article number** |
|  | DNase I  grade II, from bovine pancreas | 200 U/ml | Merk/Roche | 10104159001 |
|  | Bovine Albumin Fraction V (7.5% solution) | 1 % | Gibco™ | 15260037 |
|  | Epidermal Growth Factor from murine submaxillary gland | 20 ng/mL | Sigma-Aldrich | E4127 |
|  | Recombinant Human FGF basic (FGFb) Protein | 5 ng/mL | R&D systems | 233-FB |
| **Equipment** | **Company** | **Model/Article Number** | **CO_2_** | **Temperature** |
| **Incubation Condition** | Thermo Fischer Scientific | Heraeus® HERAcell® | 5.0% | 37 °C |
| **Equipment** | **Company** | **Model/Article Number** |  |  |
| **Centrifuge** | Hettrich Zentrifugen | Rotina 48R |  |  |
| **Cell counter** | Bio-Rad | 1450102 TC20™ Automated Cell counter |  |  |
| Nunc™ Cell Culture/Petri Dishes | Thermo Scientific™ | 172931 |  |  |
| Coating | Poly-D-lysine hydrobromide | 20 μg/mL | Sigma-Aldrich | P6407 |
| Above, media constituents and additional material needed for astrocyte differentiation from SVZ cells are provided. Abbreviations: SVZ, subventricular zone. | | | | |

| **Supplementary Table 4: Neuroinflammatory factors used** | | | | | |
| --- | --- | --- | --- | --- | --- |
| **Positive controls** | **Cytokine** | **Concentration** | **Cytokine plasma T_1/2_** | **Manufacturer** | **Article number** |
|  | IL-1α | 3 ng/ml | 2.5 minutes-1 hour | Sigma-Aldrich | I5396 |
|  | TNF-α | 30 ng/ml | 6 minutes | Cell Signaling Technology | 5178SF |
| **Neuro-inflammatory factors enriched in human TBI** | **Neuro-inflammatory factor** | **Concentration** | **Neuro-inflammatory**  **factor T_1/2_** | **Manufacturer** | **Article number** |
|  | C1Q | 400 ng/ml | 12.4 hours* | MyBioSource | #MBS143105 |
|  | C5 | 500 ng/ml | 34 minutes** | EMD Millipore | #204888 |
|  | IL-1β | 5 ng/ml | 3 minutes-4 hours* | R&D | #201-LB-005/CF |
|  | IL-6 | 10 ng/ml | 3-55 minutes* | R&D | #7270-IL-010/CF |
|  | TGF-β2 | 5 ng/ml | 2.5 hours* | R&D | #7346-B2-005/CF |
| Neuroinflammatory mediators used in the current study. T_1/2_ was derived from Refs. [2–8]  *Assessed in blood.  **Not assessed in blood; T_1/2_ of C5 convertase.  **Abbreviations:** C1Q, complement component 1Q; C5, complement component 5; IL-, interleukin-; TGF, transforming growth factor; TNF, tumor necrosis factor, T_1/2_, half-life. | | | | | |

| **Supplementary Table 5: Materials used for immunocytochemistry** | | | | | |
| --- | --- | --- | --- | --- | --- |
| **Buffers** | **Buffer type** | **Constituent** | **Concentration** | **Manufacturer** | **Article number** |
|  | Blocking buffer | PBS | 1X | Thermo Fisher Scientific | 18912-014 |
|  | Blocking buffer | Normal donkey serum | 20% | Merck Millipore | S30-100ml |
|  | Blocking buffer | Triton X-100 | 0.3% | Sigma-Aldrich | 93443 |
|  | Blocking buffer | Sodium azide | 0.1% | VWR | 103692K |
|  | Washing buffer | PBS | 1X | Thermo Fisher Scientific | 18912-014 |
|  | Washing buffer | Triton X-100 | 0.3% | Sigma-Aldrich | 93443 |
| **Primary Antibodies** | **Antibody name** | **Company** | **Article no** | **Dilution** | **Host animal** |
|  | GFAP | Abcam | Ab53554 | 1:1000 | goat |
|  | Nestin | Millipore | MAB353 | 1:100 | mouse |
|  | β -III-tubulin | Millipore | MAB1637 | 1:500 | mouse |
|  | β -III-tubulin D71G9 | Cell Signaling Technology | 5568S | 1:400 | Rabbit |
|  | AQP4 | Santa Cruz | SC-20812 | 1:100 | rabbit |
|  | vimentin | Sigma-Aldrich | V2258 | 1:500 | mouse |
|  | GLT-1 | Millipore | AB1783 | 1:200 | guinea pig |
|  | NF1A | Active Motif | 39397, 39398 | 1:500 | rabbit |
|  | P-C-Jun (Ser63) | Cell Signaling Technology | 9261L | 1:100 | rabbit |
|  | P-C-Jun (Ser73) | Cell Signaling Technology | 9164S/9164L | 1:100 | rabbit |
| **Secondary antibodies** | **Antibody name** | **Company** | **Article no** | **Dilution** | **Host animal** |
|  | Alexa 488 | Life technologies, Thermo Fisher Scientific | A21206 | 1:500 | Donkey, anti-rabbit |
|  | Alexa 488 | Life technologies, Thermo Fisher Scientific | A21202 | 1:500 | Donkey, anti-mouse |
|  | Alexa 488 | Life technologies, Thermo Fisher Scientific | A11055 | 1:500 | Donkey, anti-goat |
|  | Alexa 555 | Life technologies, Thermo Fisher Scientific | A31572 | 1:500 | Donkey, anti-rabbit |
|  | Alexa 555 | Life technologies, Thermo Fisher Scientific | A31570 | 1:500 | Donkey, anti-mouse |
|  | Alexa 555 | Life technologies, Thermo Fisher Scientific | A21432 | 1:500 | Donkey, anti-goat |
|  | Alexa 647 | Life technologies, Thermo Fisher Scientific | A31573 | 1:500 | Donkey, anti-rabbit |
|  | Alexa 647 | Life technologies, Thermo Fisher Scientific | A31571 | 1:500 | Donkey, anti-mouse |
|  | Alexa 647 | Life technologies, Thermo Fisher Scientific | A32849 | 1:500 | Donkey, anti-goat |
|  | Cy3 | Jackson ImmunoResearch | 706-165-148 | 1:500 | Donkey anti-guinea pig |
| **Nuclear marker** | **Name** | **Company** | **Article no** | **Dilution** | **Host animal** |
|  | 4’,6-diamidino-2-phenylindole dihydrochloride (DAPI) | Invitrogen | D21490 | 1:25000 | NA |
| Primary and secondary antibodies used for immunocytochemistry in the current study. **Abbreviations:** AQP4, aquaporin-4; GFAP, glial fibrillary acidic protein; GLT-1, glutamate transporter 1; NF1A, nuclear factor 1A; P-c-Jun, phosphorylated c-Jun (at serine 63 or 73); | | | | | |

| **Supplementary Table 6: Buffers used for flow-cytometry and fluorescence-activated cell sorting** | | | | |
| --- | --- | --- | --- | --- |
| **Flow cytometry buffer** | **Constituent** | **Concentration (in final solution)** | **Manufacturer** | **Article number** |
|  | Dulbecco’s phosphate buffered saline (DPBS) | 93% | Thermo Fisher Scientific | 14190169 |
|  | Glucose, 45% | 0.8% | Sigma-Aldrich | G8769 |
|  | Bovine serum albumin, 1% | 0.25% | Sigma | A2153 |
|  | N-2 | 1% | Gibco | 17502-048 |
|  | B27 | 2% | Thermo-Fisher Scientific | 17504-044 |
|  | Ethylenediaminetetraacetic acid (EDTA), 0.5M | 0.5 mM | Thermo Fisher Scientific | 15575020 |
|  | Fetal bovine serum | 2.5% | Thermo Fisher Scientific | 10082-147 |
| **Flow cytometry stainings** | **Constituent** | **Concentration** | **Manufacturer** | **Article number** |
|  | LIVE/DEAD Near-infra red Dead Cell Stain | 1:500 | Invitrogen | AC7 |
|  | Permeabilization kit, eBioscience^TM^ Foxp3/Transcription factor staining buffer set | NA | Thermo Fisher Scientific | #00-5523-00 |
|  | Ki67 | NA | BD Bioscience | V450 |
| **FACS buffer** | **Constituent** | **Concentration (in final solution)** | **Manufacturer** | **Article number** |
|  | DPBS | 95% | Thermo Fisher Scientific |  |
|  | Glucose, 45% | 0.8% | Sigma-Aldrich | G8769 |
|  | Bovine serum albumin, 1% | 0.25% | Sigma | A2153 |
|  | N-2 | 1% | Gibco | 17502-048 |
|  | B27 | 2% | Thermo-Fisher Scientific | 17504-044 |
|  | Ethylenediaminetetraacetic acid (EDTA), 0.5M | 1 mM | Thermo Fisher Scientific | 15575020 |
|  | Fetal bovine serum | 1% | Thermo Fisher Scientific | 10082-147 |
| Above, buffer constituents and additional material needed for flow-cytometry or fluorescence-activated cell sorting media are provided. Abbreviations: FACS, fluorescence-activated cell sorting. | | | | |

Supplementary Figures and Supplementary Figure Legends


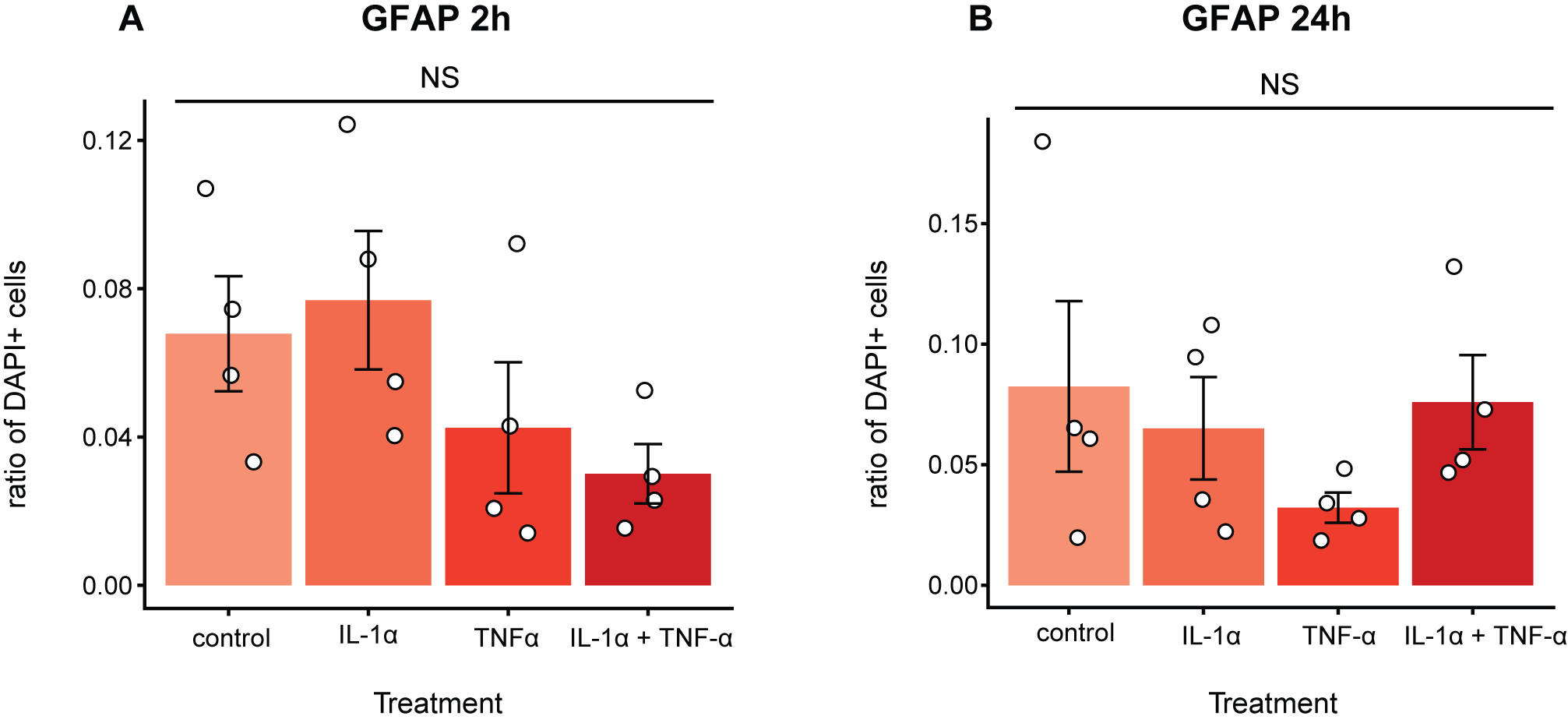


**Supplementary Figure 1: Glial fibrillary acidic protein is not an optimal marker of ES-astrocyte activation induced by IL-1 α and TNF- α.**

ES-astrocyte activation induced by the cytokines IL-1 α and TNF- α conferred a high increase in the phosphorylation of c-Jun but not of GFAP either at 2 hours of stimulation **(A)** or at 24 hours of stimulation **(B)**. **Abbreviations:** ES, embryonic stem cell-derived; GFAP, glial fibrillary acidic protein; IL-, interleukin; NS, non-significant; TNF, tumor necrosis factor.


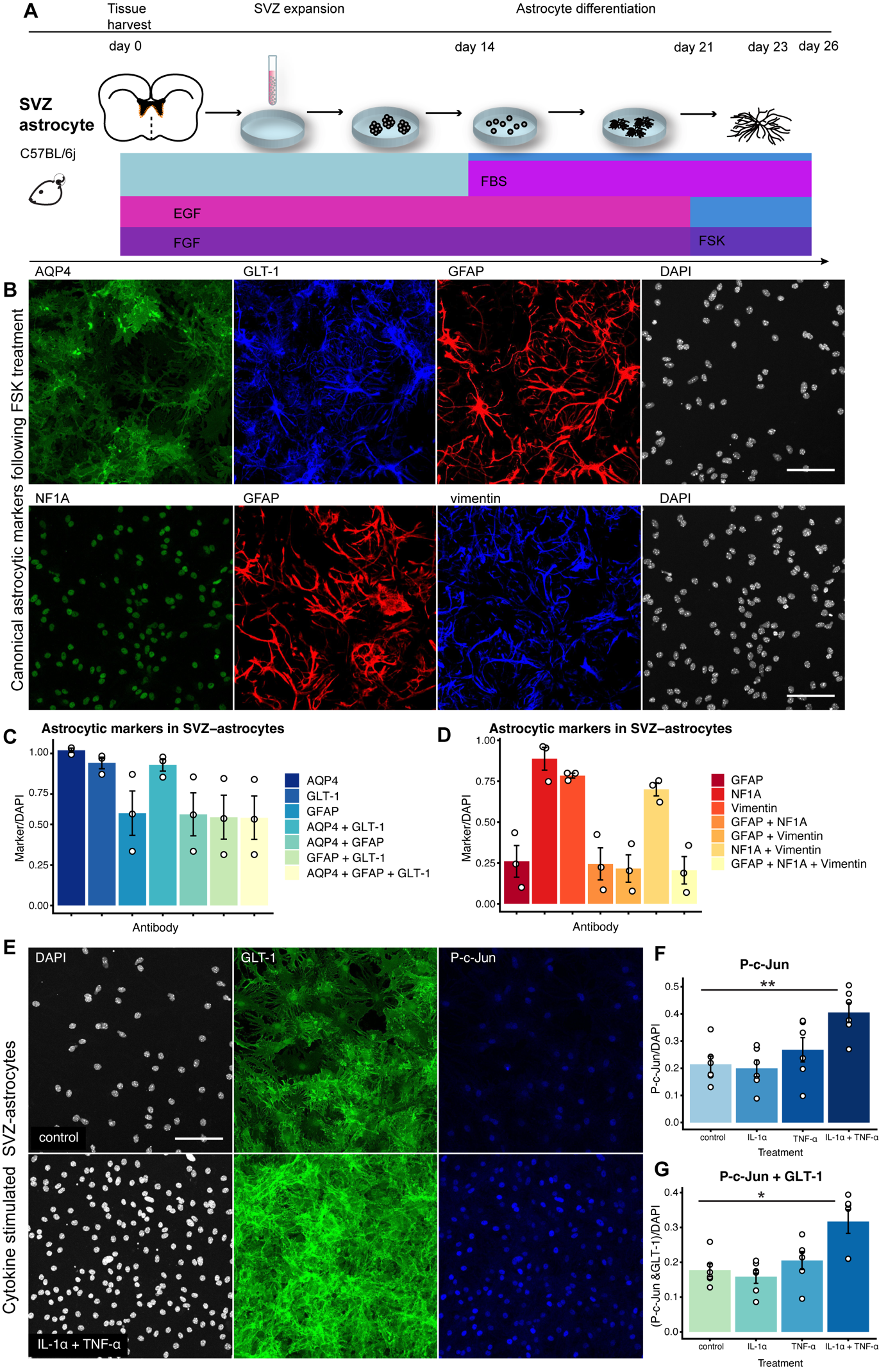


**Supplementary Figure 2: Primary culture validation in subventricular zone-astrocytes.**

We validated our culture protocols using subventricular zone (SVZ) cells that we differentiated into astrocytes **(A)**. The SVZ-derived astrocytes expressed numerous canonical astrocytic markers at the protein level **(B)**. Of note, the majority of markers were expressed in >75% of cells with consequent high extent of co-localization **(C, D)**. Following cytokine exposure **(E)**, P-c-Jun was significantly upregulated **(F)**, and the cell phenotype of P-c-Jun activated cells was astrocytic **(G)**. **Significance level:** *, p ≤ 0.05; **, p ≤ 0.01. **Abbreviations:** AQP4, aquaporin-4; DAPI, 4’,6-diamidino-2-phenylindole dihydrochloride; EGF epidermal growth factor; FBS, fetal bovine serum; FGF, fibroblast growth factor; FSK, forskolin; GFAP, glial fibrillary acidic protein; GLT-1, glutamate transporter 1; IL-1α, interleukin 1α; NF1A, nuclear factor 1A; P-c-Jun, phosphorylated c-Jun; SVZ, sub ventricular zone; TNF-α, tumor necrosis factor α. **Scale bars**: 100 μm.


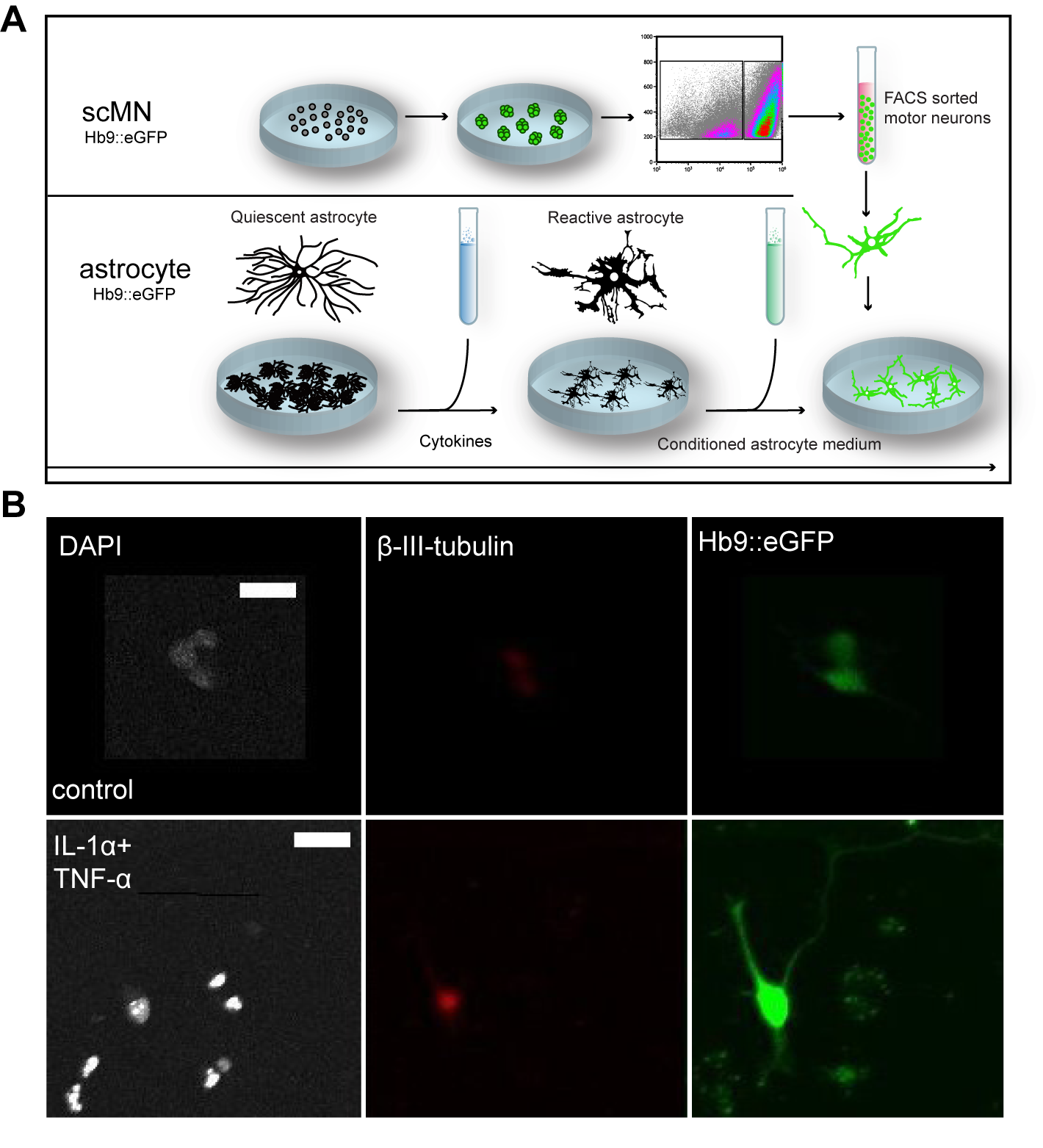


**Supplementary Figure 3: Contact-independent co-culture system of astrocyte-mediated neurotoxicity.**

We assessed whether the observed neurotoxic effect was contact-dependent or cell-autonomous by cultivating motor neurons individually, and by transferring conditioned (non-concentrated) reactive astrocyte medium to them **(A)**. Cell survival was very low **(B)**, indicating that astrocytic trophic support is pivotal following FACS sorting. **Abbreviations:** DAPI, 4’,6-diamidino-2-phenylindole dihydrochloride; FACS, fluorescence activated cell sorting; eGFP, enhanced green fluorescent protein; Hb9, homeobox Hb9; IL-1α, interleukin 1α; sc, spinal cord/brainstem; TNF-α, tumor necrosis factor α. **Scale bars**: **B**, 25 μm.


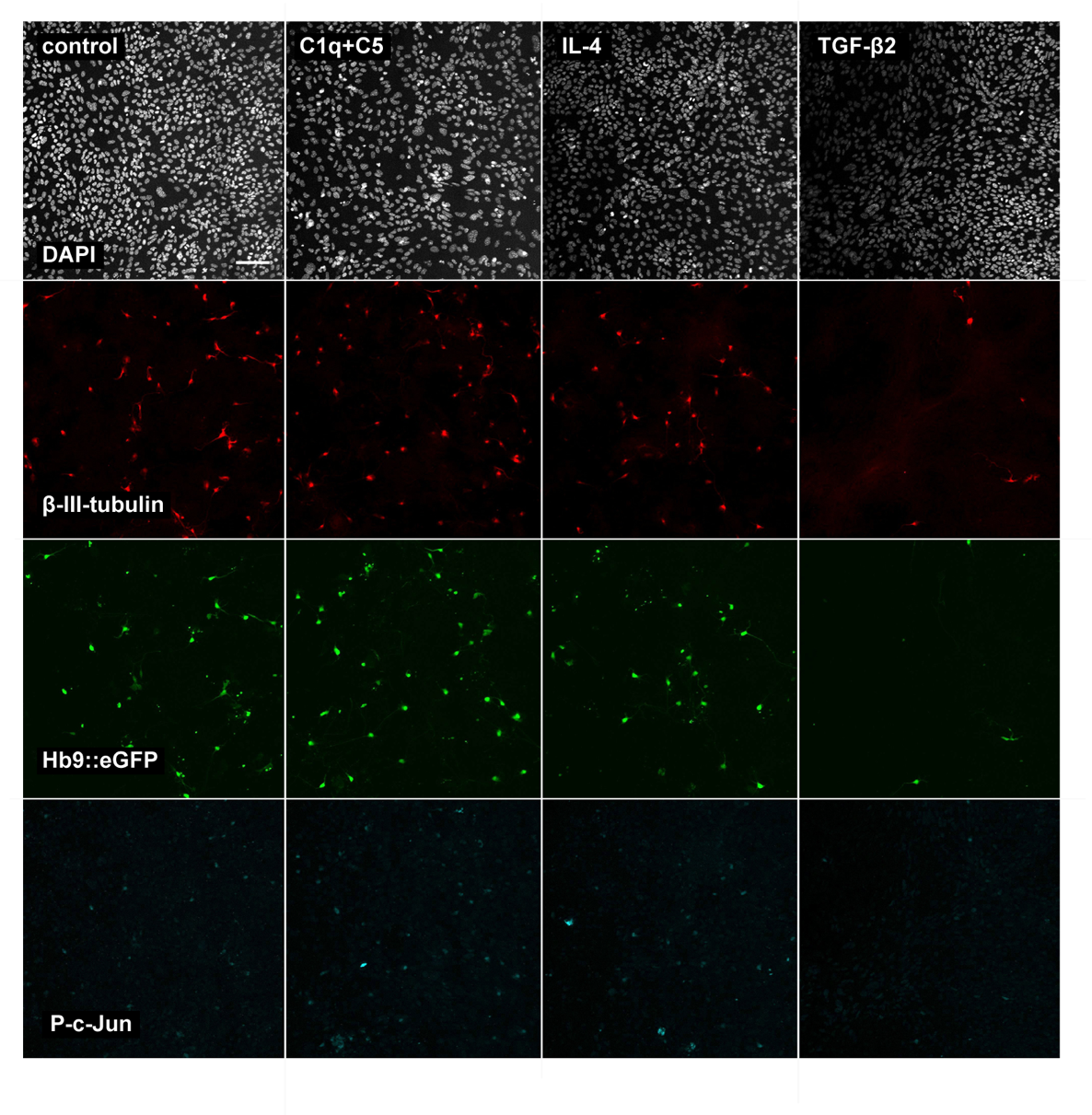


**Supplementary Figure 4: Immunocytochemical depiction of translationally relevant neuroinflammatory mediators.** ES-derived astrocytes were co-cultured with motor neurons following stimulation with a panel of neuroinflammatory mediators recently described by us [9]. **Scale bar**: 100 μm. **Abbreviations:** C, complement component; DAPI, 4’,6-diamidino-2-phenylindole dihydrochloride; eGFP, enhanced green fluorescent protein; Hb9, homeobox Hb9; IL-4, interleukin 4; P-c-Jun, phosphorylated c-Jun; TGF-β2, transforming growth factor β 2.

**Supplementary References**

1. Edgar R, Domrachev M, Lash AE. Gene Expression Omnibus: NCBI gene expression and hybridization array data repository. Nucleic Acids Res. 2002;30:207–10.

2. Poole S, Bird TA, Selkirk S, Gaines-Das RE, Choudry Y, Stephenson SL, et al. Fate of injected interleukin 1 in rats: Sequestration and degradation in the kidney. Cytokine. 1990;2:416–22.

3. Beutler BA, Milsark IW, Cerami A. Cachectin/tumor necrosis factor: production, distribution, and metabolic fate in vivo. J Immunol [Internet]. 1985;135:3972–7. Available from: http://www.ncbi.nlm.nih.gov/pubmed/2999236

4. Veerhuis R, Van Es LA, Daha MR. In vivo degradation of rat C1q induced by intravenous injection of soluble IgG aggregates. Immunology. 1985;54:801–80110.

5. DiScipio RG. The conversion of human complement component C5 into fragment C5b by the alternative-pathway C5 convertase. Biochemical Journal. 1981;199:497–504.

6. McLennan IS, Weible MW, Hendry IA, Koishi K. Transport of transforming growth factor-β2 across the blood-brain barrier. Neuropharmacology. 2005;48:274–82.

7. CASTELL J V., GEIGER T, GROSS V, ANDUS T, WALTER E, HIRANO T, et al. Plasma clearance, organ distribution and target cells of interleukin‐6/hepatocyte‐stimulating factor in the rat. Eur J Biochem. 1988;177:357–61.

8. Klapproth J, Castell J, Geiger T, Andus T, Heinrich PC. Fate and biological action of human recombinant interleukin 1β in the ratin vivo. Eur J Immunol [Internet]. 1989;19:1485–90. Available from: https://onlinelibrary.wiley.com/doi/10.1002/eji.1830190821

9. Lindblad C, Pin E, Just D, Al Nimer F, Nilsson P, Bellander B-M, et al. Fluid Proteomics of CSF and Serum Reveal Important Neuroinammatory Proteins in Blood-Brain Barrier Disruption and Outcome Prediction Following Severe Traumatic Brain Injury: A Prospective, Observational Study. Crit Care [Internet]. BioMed Central; 2021;1–28. Available from: https://doi.org/10.21203/rs.3.rs-96625/v1
