# Supplementary figures and images for: Stem cell-derived brainstem mouse astrocytes obtain a neurotoxic phenotype in vitro upon neuroinflammation
Source: J Inflamm (Lond). 2023 Jun 27;20:22. doi: 10.1186/s12950-023-00349-8 (PMC10303821; doi:10.1186/s12950-023-00349-8)

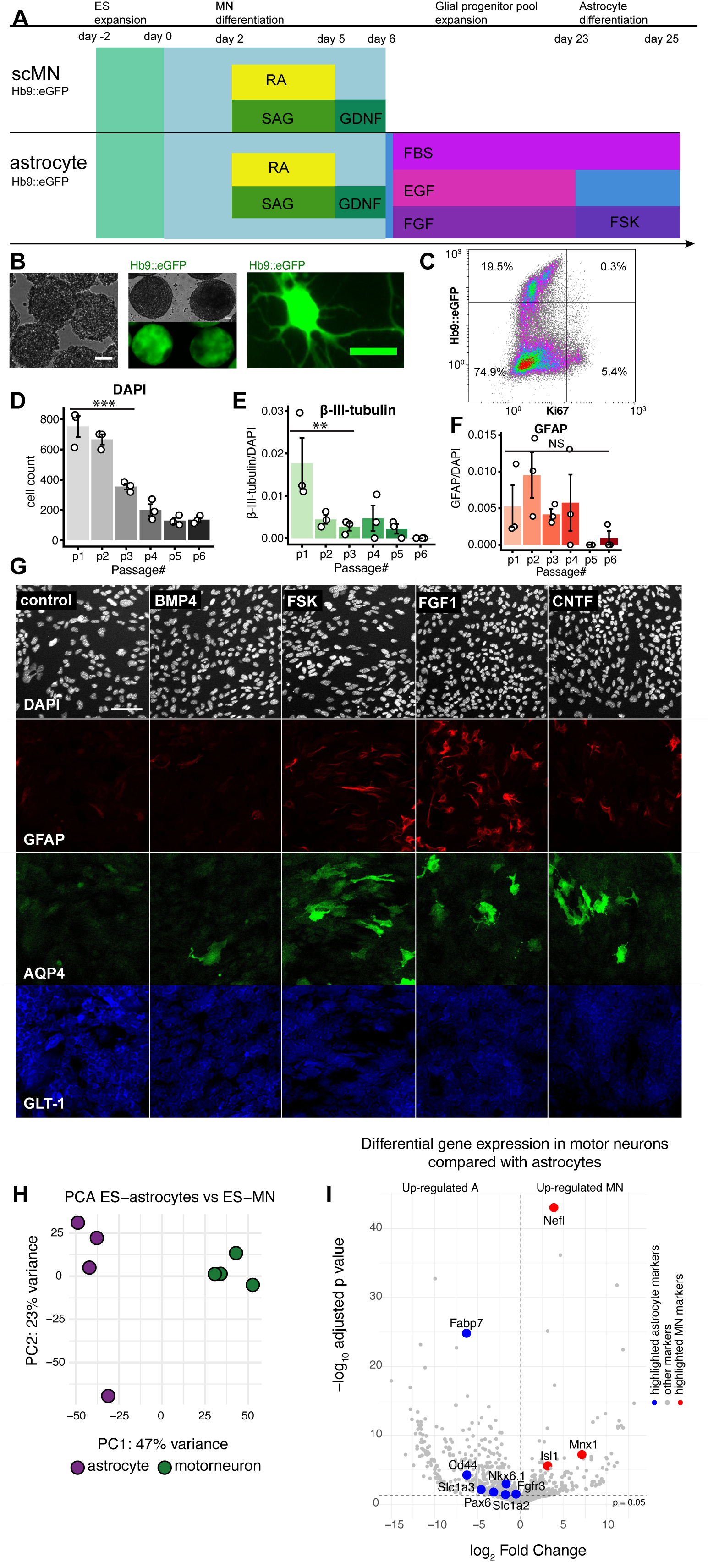

Supplement: Supplementary file 1 — Additional file 1: Supplementary Methods; Supplementary References. Supplementary Table 1. Media constituents for motor neuron differentiation. Supplementary Table 2. Media constituents for astrocyte differentiation. Supplementary Table 3. Media constituents for primary SVZ cell culture. Supplementary Table 4. Neuroinflammatory factors used. Supplementary Table 5. Materials used for immunocytochemistry. Supplementary Table 6. Buffers used for flow-cytometry and fluorescence-activated cell sorting. Supplementary Figure 1. Glial fibrillary acidic protein is not an optimal marker of ES-astrocyte activation induced by IL-1 α and TNF- α. Supplementary Figure 2. Primary culture validation in subventricular zone-astrocytes. Supplementary Figure 3. Contact-independent co-culture system of astrocyte-mediated neurotoxicity. Supplementary Figure 4. Immunocytochemical depiction of translationally relevant neuroinflammatory mediators. [file 12950_2023_349_MOESM1_ESM.zip › figure1_230622_CL.tif]

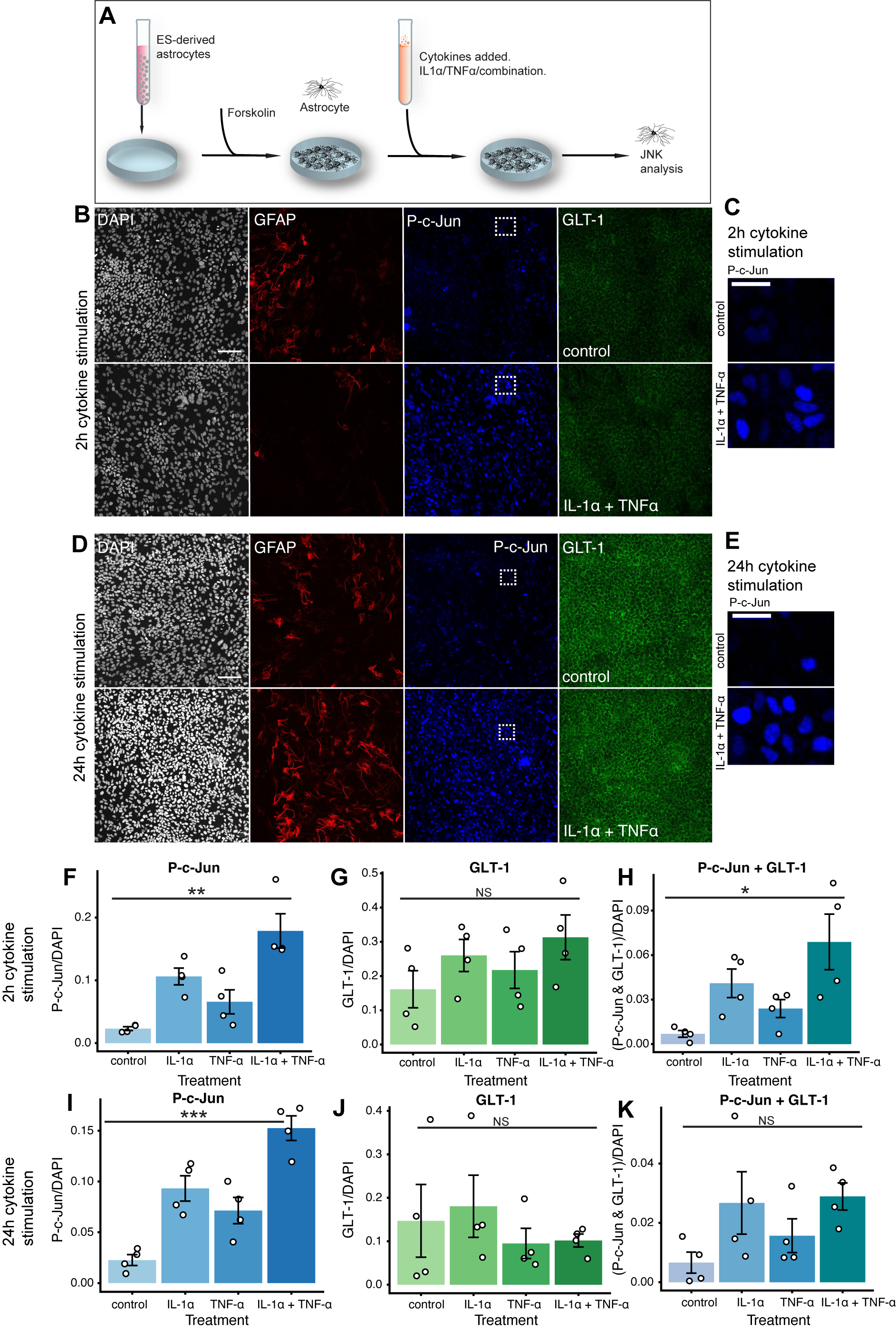

Supplement: Supplementary file 1 — Additional file 1: Supplementary Methods; Supplementary References. Supplementary Table 1. Media constituents for motor neuron differentiation. Supplementary Table 2. Media constituents for astrocyte differentiation. Supplementary Table 3. Media constituents for primary SVZ cell culture. Supplementary Table 4. Neuroinflammatory factors used. Supplementary Table 5. Materials used for immunocytochemistry. Supplementary Table 6. Buffers used for flow-cytometry and fluorescence-activated cell sorting. Supplementary Figure 1. Glial fibrillary acidic protein is not an optimal marker of ES-astrocyte activation induced by IL-1 α and TNF- α. Supplementary Figure 2. Primary culture validation in subventricular zone-astrocytes. Supplementary Figure 3. Contact-independent co-culture system of astrocyte-mediated neurotoxicity. Supplementary Figure 4. Immunocytochemical depiction of translationally relevant neuroinflammatory mediators. [file 12950_2023_349_MOESM1_ESM.zip › figure2_230622_CL.tif]

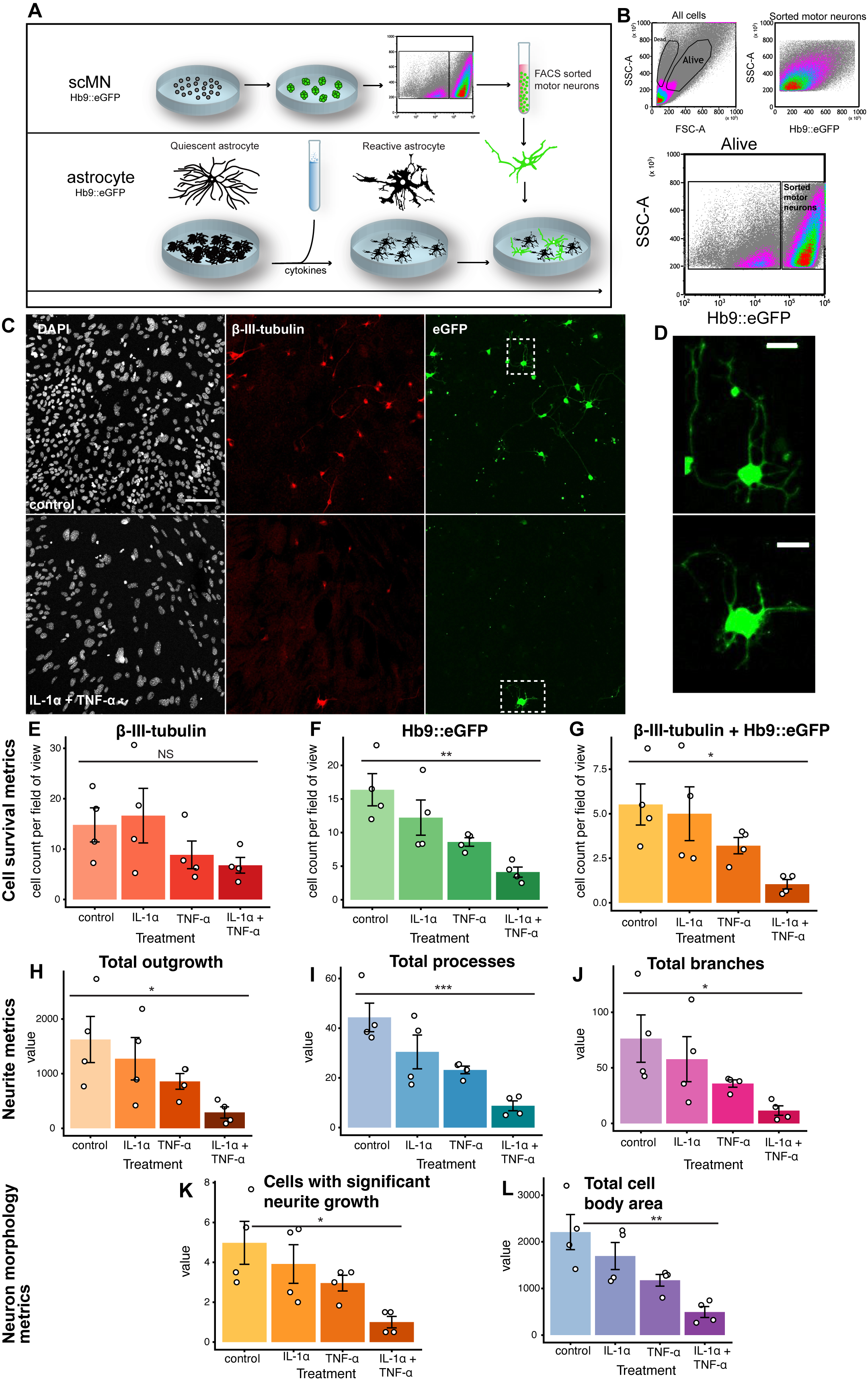

Supplement: Supplementary file 1 — Additional file 1: Supplementary Methods; Supplementary References. Supplementary Table 1. Media constituents for motor neuron differentiation. Supplementary Table 2. Media constituents for astrocyte differentiation. Supplementary Table 3. Media constituents for primary SVZ cell culture. Supplementary Table 4. Neuroinflammatory factors used. Supplementary Table 5. Materials used for immunocytochemistry. Supplementary Table 6. Buffers used for flow-cytometry and fluorescence-activated cell sorting. Supplementary Figure 1. Glial fibrillary acidic protein is not an optimal marker of ES-astrocyte activation induced by IL-1 α and TNF- α. Supplementary Figure 2. Primary culture validation in subventricular zone-astrocytes. Supplementary Figure 3. Contact-independent co-culture system of astrocyte-mediated neurotoxicity. Supplementary Figure 4. Immunocytochemical depiction of translationally relevant neuroinflammatory mediators. [file 12950_2023_349_MOESM1_ESM.zip › figure3_230622_CL.tif]

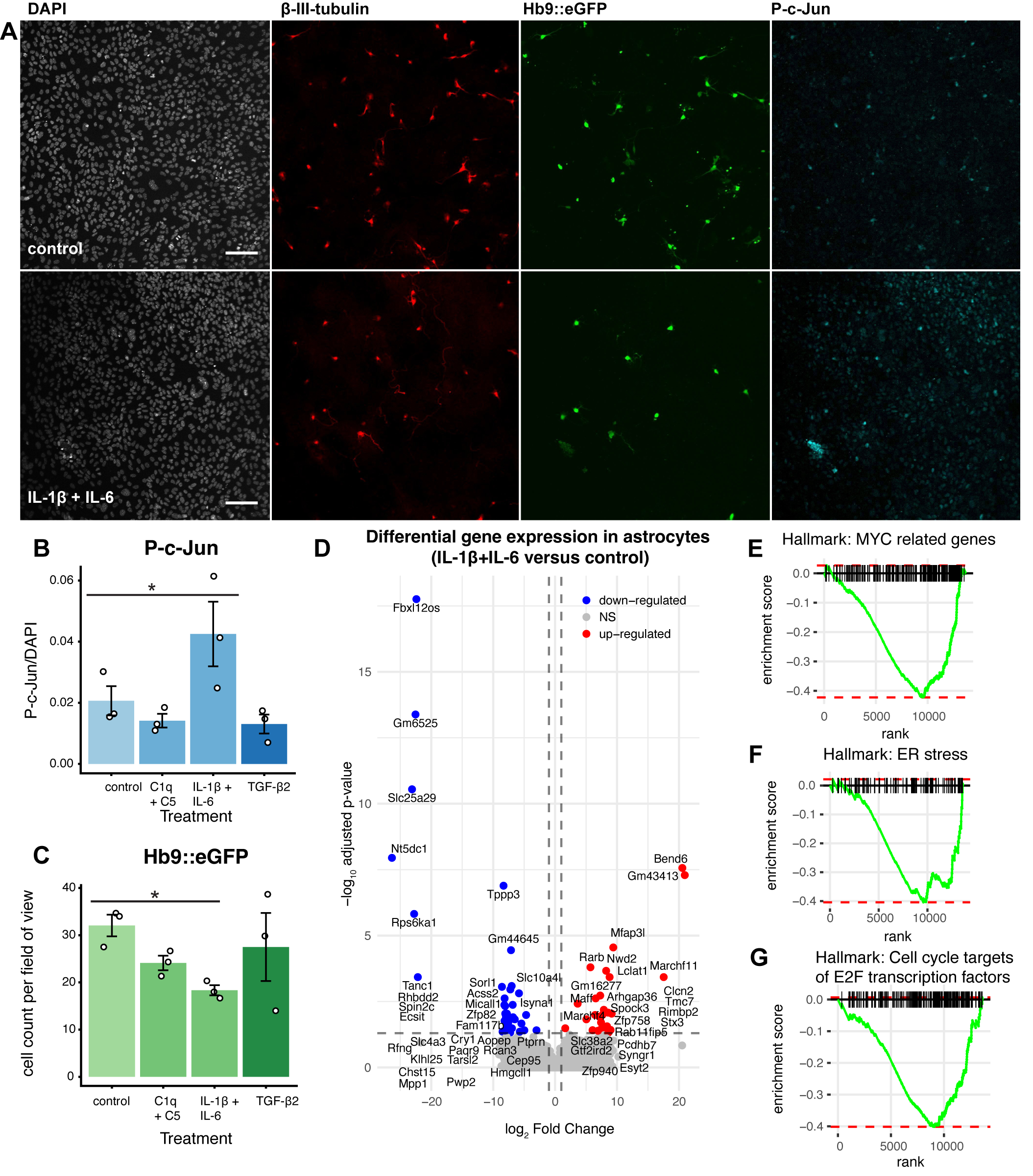

Supplement: Supplementary file 1 — Additional file 1: Supplementary Methods; Supplementary References. Supplementary Table 1. Media constituents for motor neuron differentiation. Supplementary Table 2. Media constituents for astrocyte differentiation. Supplementary Table 3. Media constituents for primary SVZ cell culture. Supplementary Table 4. Neuroinflammatory factors used. Supplementary Table 5. Materials used for immunocytochemistry. Supplementary Table 6. Buffers used for flow-cytometry and fluorescence-activated cell sorting. Supplementary Figure 1. Glial fibrillary acidic protein is not an optimal marker of ES-astrocyte activation induced by IL-1 α and TNF- α. Supplementary Figure 2. Primary culture validation in subventricular zone-astrocytes. Supplementary Figure 3. Contact-independent co-culture system of astrocyte-mediated neurotoxicity. Supplementary Figure 4. Immunocytochemical depiction of translationally relevant neuroinflammatory mediators. [file 12950_2023_349_MOESM1_ESM.zip › figure4_230622_CL.tif]
